# Perinatal Western‐style diet exposure associated with altered sensory functional connectivity in infant Japanese macaques

**DOI:** 10.14814/phy2.70674

**Published:** 2025-12-02

**Authors:** Samantha Papadakis, Eric Feczko, Julian S. B. Ramirez, Oscar Miranda‐Dominguez, Darrick Sturgeon, Thomas J. Madison, Anders J. Perrone, Eric Earl, AJ Mitchell, Geoffrey A. Dunn, Elinor L. Sullivan, Damien A. Fair

**Affiliations:** ^1^ Department of Behavioral Neuroscience Oregon Health & Science University Portland Oregon USA; ^2^ Department of Psychiatry Oregon Health & Science University Portland Oregon USA; ^3^ Department of Pediatrics University of Minnesota Medical School Minneapolis Minnesota USA; ^4^ Masonic Institute for the Developing Brain University of Minnesota Medical School Minneapolis Minnesota USA; ^5^ Division of Neuroscience, Oregon National Primate Research Center Oregon Health & Science University Beaverton Oregon USA; ^6^ Department of Human Physiology University of Oregon Eugene Oregon USA; ^7^ Institute of Child Development, College of Education and Human Development University of Minnesota Minneapolis Minnesota USA

**Keywords:** amygdala, motor cortex, neurodevelopment, nonhuman primates, sensory processing, somatosensory

## Abstract

Sensory processing disorder (SPD) is a neurodevelopmental condition characterized by impaired sensory discrimination and responsivity. Although the causes and neural correlates of SPD remain poorly understood, prenatal influences should be considered, as the prenatal environment is strongly implicated in the progression of neurodevelopmental disorders. One factor hypothesized to promote SPD is perinatal Western‐style diet (WSD) exposure. This study explored the effects of perinatal WSD exposure on the proposed neural correlates of SPD in Japanese macaques. Functional connectivity between sensory and emotional processing areas was assessed at 4 months of age using resting‐state functional magnetic resonance imaging (rs‐fMRI). A machine learning model successfully predicted perinatal diet group based on functional connectivity strengths, indicating that differences in sensory connectivity exist between diet groups. Intra‐somatomotor, visual‐auditory, somatomotor‐auditory, somatomotor‐visual, and intra‐visual network connections demonstrated the greatest differences between groups, with primary motor cortex connectivity being the most impacted. Connections to the amygdala were not major contributors to accurate model performance, but amygdala connectivity, especially to the somatomotor network, may still be a weak driver of model performance. These findings suggest that a proposed predictor of SPD, perinatal WSD exposure, impacts the functional connectivity of sensory processing areas relevant in SPD during early infancy.

## Graphical abstract

Perinatal Western‐style diet exposure was associated with altered functional connectivity in sensory and emotional processing areas in 4‐month‐old macaques.

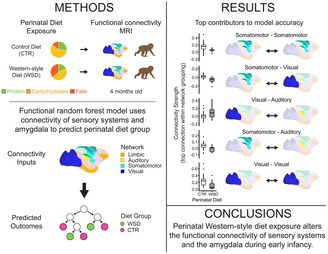

## 1 INTRODUCTION

Sensory processing disorders (SPD) impact roughly 14% of children in the US (Ahn et al., 2004), yet the etiology of this condition remains poorly understood. Although many children with SPD do not have a comorbid psychiatric diagnosis, approximately 95% of children with autism spectrum disorder (ASD) report a SPD (Crane et al., 2009; Leekam et al., 2007; Tomchek & Dunn, 2007), indicating that there may be some overlap between the conditions that lead to ASD and those that lead to SPD. Many genetic and environmental factors have been linked to the development of ASD, including the state of the maternal environment during gestation (Love et al., 2024; Wei et al., 2024). Three highly correlated maternal factors have been shown to increase the incidence of ASD behaviors in offspring: the consumption of a Western‐style diet (WSD) that is high in saturated fats and sugar, obesity, and an elevated inflammatory state (Careaga et al., 2017; DeCapo et al., 2019; Fernandes et al., 2021; Gawlińska et al., 2021; Guma et al., 2019; Howard et al., 2011; Parker‐Athill & Tan, 2010). Animal studies commonly use a WSD to induce obesity and the associated inflammatory state in dams in order to study impacts on offspring neurodevelopment that are characteristic of the behaviors and phenotypes of many neurodevelopmental disorders, including ASD (Peleg‐Raibstein et al., 2012; Sullivan et al., 2010). However, more research is needed to determine whether prenatal WSD exposure is associated with SPD symptoms, as well.

SPD is a broad umbrella term for conditions that affect sensory processing, including sensory modulation disorder (SMD), sensory discrimination disorder (SDD), and sensory‐based motor disorder (SBMD) (Miller et al., 2007; Mulligan et al., 2021). Although the symptoms of these disorders are well described, the neural correlates that give rise to them are poorly understood. A handful of studies have explored neural connectivity at the structural and functional level. Diffusion tensor imaging (DTI) has demonstrated substantially decreased white matter microstructural integrity in primary sensory cerebral tracts and pathways involved in multisensory integration in boys with SPD between 8 and 11 years old (Owen et al., 2013). Altered functional connectivity was found in children between 9 and 12 years old with sensory over‐responsivity (SOR), a subtype of SPD, as well (Schwarzlose et al., 2023). Impacted areas included sensorimotor and visual networks, as well as connections to non‐sensory areas including the hippocampus and amygdala. Amygdala connectivity could explain the negative emotions that arise with SOR, and there is evidence to suggest that aberrant connections from sensory processing pathways to the amygdala may be implicated in individuals with ASD that experience symptoms of SOR (Møller et al., 2005). In addition to identifying the aberrant connections that are prominent in SPD, further research is needed to elucidate the mechanisms that form these connections. Microglia, for example, shape neural circuitry by controlling neurogenesis during the third trimester and, later, by actively pruning synapses in early childhood (Cunningham et al., 2013; Eltokhi et al., 2020). Prenatal inflammation would therefore impact neural circuitry through a different microglial‐mediated mechanism than postnatal inflammation, leading to potentially different outcomes. An increased understanding of how these processes contribute to the effects seen in SPD could help direct targeted therapeutic approaches.

These findings illustrate that sensory and amygdala connectivity may be altered in children with SPD, but it is still unclear which prenatal factors may increase the likelihood of these outcomes. Additionally, these limited SPD studies have only examined differences during the preadolescent period. A comprehensive exploration of changes in connectivity across development could help identify markers for early detection of SPD and contribute to the understanding of how brain networks are remodeled over time. Thus, the field of SPD research would benefit from further investigation of prenatal predictors and longitudinal study designs. Animal models are better suited for these design elements, but they are often limited by their sample size and narrow behavioral measurements. As a result, the findings are not directly translatable or as clinically relevant as those from human studies. One way to preserve relevancy is to use non‐human primate (NHP) models, as NHPs display highly conserved attributes including complex behavior, brain organization, gestational and neurodevelopmental timelines, placental structure and function, and metabolic and immune functioning (Carter, 2007; Estes et al., 2018; Miranda‐Dominguez et al., 2014; Sullivan & Kievit, 2016).

The present study addresses these species‐specific gaps by examining the effects of a proposed predictor of SPD—perinatal WSD exposure—on proposed neural correlates of SPD in a highly conserved NHP model. Resting‐state functional magnetic resonance imaging (rs‐fMRI) scans were collected in 4‐month‐old Japanese macaques to measure the strength of functional connections within the brain. A set of connections between the sensory networks and the amygdala was selected to probe for differences in the areas that are hypothesized to be involved in SPD and SOR. Differences in connectivity successfully trained a machine learning model to classify NHP subjects by perinatal diet group. The features that drove accurate model prediction were evaluated to identify the brain connections that were most impacted by perinatal WSD exposure. Supplemental and preliminary analyses engaged a longitudinal study design to explore whether functional connectivity differences persisted across development at 6, 11, 21, and 36 months of age. Additionally, differences in connectivity at the latest time point, 36 months, were used to predict two other measures: (1) maternal adiposity, to determine whether increased adiposity would have a separate impact from perinatal WSD exposure, and (2) a concurrent measure of postnatal neuroinflammation in the offspring—the number of microglia within the amygdala—to probe for an association between altered connectivity and a potential mediator of altered connectivity within individuals. None of these measures were successfully predicted from connectivity, indicating no long‐term effects associated with perinatal diet, maternal adiposity, or postnatal neuroinflammation. A final supplemental analysis sought to provide greater translational value by determining whether the impacts on brain connectivity in NHPs directly translated to the functional connectivity profile observed in a larger sample of children with SOR. Although the machine learning model was unable to predict SOR from the same set of sensory and amygdala connections in 9‐ and 10‐year‐old children, this was consistent with the null findings from the NHPs at the same developmental age (36 months). These results highlight the presence of strong impacts on sensory and emotional processing areas during early infancy and hint at the potential for recovery over time.

## 2 MATERIALS AND METHODS

### 2.1 Animal model

A well‐established NHP model of WSD‐induced obesity was chosen for this study (Sullivan et al., 2010, 2012). In this model, dams were fed either a WSD or a control diet (CTR). A larger proportion of dams on the WSD developed obesity, defined as >19.6% body fat pre‐pregnancy, though some dams on the CTR diet spontaneously developed obesity as well (McCurdy et al., 2009). As these macaques demonstrate the full range of human metabolism, this natural variation in weight gain is consistent with what is observed in humans (Thompson et al., 2017, 2018).

Importantly, the offspring of dams that consumed a WSD have demonstrated the component behaviors of multiple neurodevelopmental disorders, including ASD. These behaviors include increased anxiety and aggression (Sullivan et al., 2010; Thompson et al., 2017, 2018), decreased social engagement, and increased idiosyncratic behaviors consisting of abnormal movement, abnormal posture, increased head tossing, and repetitive stereotypy (Mitchell et al., 2022).

Additional maternal and offspring phenotypes from this model, including inflammatory profiles and neuronal impacts, have been characterized in earlier reports (Comstock et al., 2012; Dunn, Mitchell, et al., 2022; Dunn, Thompson, et al., 2022; Grayson et al., 2010; McCurdy et al., 2009; Mitchell et al., 2022; Papadakis, 2023; Papadakis et al., 2024; Ramirez et al., 2020, 2021; Sullivan et al., 2010, 2017; Thompson et al., 2018).

All animal group demographic measures are presented for the set of 39 subjects that were included in the 4‐month‐old analyses in the present study; see Table S1 for demographics relating to the full cohort of subjects, including those scanned at 6, 11, 21, and 36 months of age for the supplemental analyses.

All animal procedures were in accordance with National Institutes of Health guidelines on the ethical use of animals and were approved by the Oregon National Primate Research Center (ONPRC) Institutional Animal Care and Use Committee.

### 2.2 Macaque dietary information

The experimental diets varied in their energy sources, with the WSD deriving a greater proportion of calories from fat. The CTR diet (Monkey Diet no. 5000; Purina Mills) provided approximately 14.7% of calories from fat, 58.5% from carbohydrates, and 26.8% from protein. The WSD (TAD Primate Diet no. 5L0P, Test Diet; Purina Mills) provided approximately 36.6% of calories from fat, 45.0% from carbohydrates, and 18.4% from protein. The chemical composition of the diets also varied, with the WSD containing a larger proportion of fats and sugars. Saturated fat comprised approximately 0.9% of the CTR diet formulation and 5.4% of the WSD. Monounsaturated and polyunsaturated fats comprised 4.4% of the CTR diet and 9.0% of the WSD. Sugars comprised approximately 3.1% of the CTR diet and 18.9% of the WSD, and they were primarily present as fructose and sucrose. The WSD group was also provided with calorically dense treats (35.7% of calories from fat, 56.2% from carbohydrates, and 8.1% from protein) on a daily basis. The macronutrient composition was obtained from diet specification sheets and has been reported in greater detail previously (Thompson et al., 2017).

### 2.3 Adult female macaques

Adult Japanese macaques (*Macaca fuscata*) were housed in indoor/outdoor pens that each contained between 4 and 12 individuals, with a male/female group ratio of 1–2/3–10. Animals were randomly assigned to either the CTR or WSD at least 14 months prior to offspring birth. Both groups were provided ad libitum access to the diet and water, and each received fruits and vegetables for daily nutritional enrichment. Pregnant females underwent sedation two to three times for fetal dating and third trimester measures before giving birth naturally in their social groups. Mean maternal age at offspring birth was 11.31 years (SD 3.61) for the CTR group and 9.40 years (SD 2.28) for the WSD group. Mean maternal pre‐pregnancy weight was 10.25 kg (SD 1.96) for the CTR group and 11.33 kg (SD 1.37) for the WSD group, with one missing value for one of the WSD offspring.

### 2.4 Macaque juvenile offspring

Juvenile subjects were born over the course of seven consecutive years. Offspring began consuming the maternal diet as early as 4 months of age, and by 6 months it became their primary food source. This continued exposure to the assigned diet before weaning is therefore better described as a perinatal exposure rather than a purely prenatal exposure. Offspring were weaned at a mean age of 7.10 months (SD 1.99) for the CTR group and 7.35 months (SD 1.50) for the WSD group (6 and 3 missing values for the CTR and WSD groups, respectively). This study included maternal siblings (21 of the 39 subjects scanned at 4 months of age had at least one sibling in the study, with all 21 born from a total of 10 dams), though paternal identification was unknown.

A total of 81 juveniles were selected for this study. After excluding subjects for medical issues, a change in maternal diet partway through gestation, and poor quality scans at every available age point, 69 juveniles remained in the study. Of these, 39 contributed acceptable‐quality scans at 4 months of age; quality control criteria are described below. This study aimed to balance for perinatal diet group and offspring sex (*N* = 39; CTR *n* = 22, female *n* = 16; WSD *n* = 17, female *n* = 3). However, to increase the sample size for use with functional connectivity analyses, this study combined scans that were collected under two separate acquisition protocols, as the parameter settings for the resting‐state functional scans were identical. This factor was not accounted for as a covariate in the classification analysis. While this presents a limitation, prior work in this model has established that the change in scan acquisition protocol does not have a significant effect on mean cortical thickness across the time points included in the study (Ramirez et al., 2021). This addition increased the sample size to a quantity that is expected to be sufficient for small‐scale neuroimaging‐only analyses, as previous work has demonstrated that small samples with as few as 25 subjects can be useful for representing central tendencies of functional brain organization among groups, especially with longitudinal study designs (Marek et al., 2022).

### 2.5 Macaque subject demographics

**FIGURE 1:**
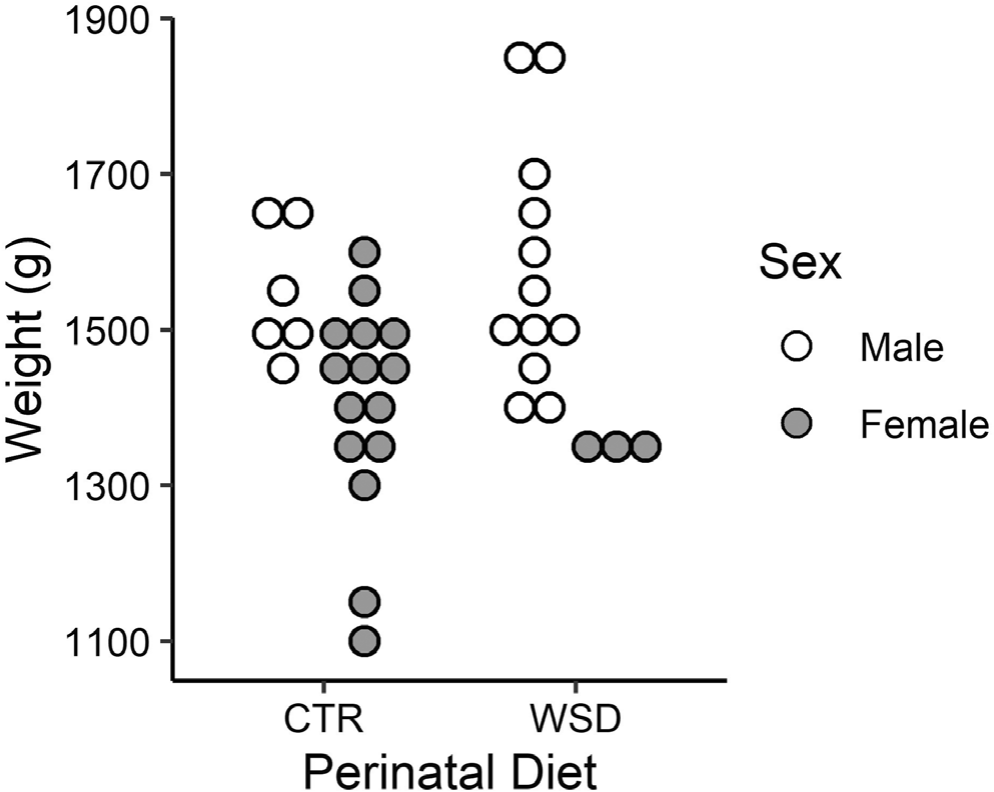
Macaque offspring weight at 4 months of age. Offspring weight (*N* = 36, female *n* = 18) is displayed in grams. Subjects are clustered by perinatal diet group and sex, with males and females represented by white and gray markers, respectively. CTR, control diet; WSD, Western‐style diet.

MRI scans were acquired in offspring at a mean age of 4.41 months (SD 0.15) at the time of the scan. The demographic composition of the 4‐month age group, including the number of subjects scanned under each acquisition protocol, is summarized in Table 1. The weight of each subject at the time of the scan is displayed in Figure 1. Mean offspring weight was 1.44 kg (SD 0.14) for the CTR group and 1.53 kg (SD 0.17) for the WSD group (1 and 2 missing values for the CTR and WSD groups, respectively).

**TABLE 1:** Demographic composition of macaque subjects at 4 months of age.

Subjects in 4‐month group				
Scan protocol	#1		#2	
Perinatal diet	CTR	WSD	CTR	WSD
Male	3	9	3	5
Female	7	2	9	1
Total	39			

Abbreviations: CTR, control diet; WSD, Western‐style diet.

### 2.6 Macaque MRI acquisition

The MRI acquisition protocols have been previously described in prior publications from the research group (Ramirez, 2019; Ramirez et al., 2020, 2021).

Imaging was obtained in a single session for each macaque subject at each time point. MRI scans were acquired on a Siemens TIM Trio 3.0 Tesla scanner. A 15‐channel knee coil was modified to scan the heads of the macaque offspring. Ketamine (10–15 mg/kg) was administered to allow for intubation, and macaques were maintained on <1.5% isoflurane anesthesia for the duration of the scan. Macaques were continuously monitored for irregularities in heart rate, respiration, and peripheral oxygen saturation.

The scan acquisition protocol was updated partway through the study to improve image quality. Importantly, the acquisition parameters for the resting‐state functional scan were not changed. The acquisition parameters for protocols #1 and #2 are as follows. Both protocols acquired a total of four T1‐weighted (T1w) anatomical images (Scan Protocol #1: TE = 3.86 ms, TR = 2500 ms, TI = 1100 ms, flip angle = 12°, 0.5 mm isotropic voxel; Scan Protocol #2: TE = 3.33 ms, TR = 2600 ms, TI = 900 ms, flip angle = 8°, 0.5 mm isotropic voxel), which were averaged to improve the signal‐to‐noise ratio. Both protocols acquired one T2‐weighted (T2w) anatomical image (Scan Protocol #1: TE = 95 ms, TR = 10,240 ms, flip angle = 150°, 0.5 mm isotropic voxel; Scan Protocol #2: TE = 407 ms, TR = 3200 ms, 0.5 mm isotropic voxel). Both protocols acquired a 30‐minute resting‐state blood oxygen level dependent (BOLD) scan using the same acquisition settings. This scan was acquired 45 min after the initial ketamine injection and utilized a gradient echo‐planar imaging (EPI) sequence (TR = 2070 ms, TE = 25 ms, FA = 90°, 1.5 mm^3^ voxels, 32 slices with interleaved acquisition, FOV = 96 × 96 mm). For distortion correction purposes, Scan Protocol #1 acquired field map images (TR = 450 ms, TE = 5.19 ms/7.65 ms, FA = 60°, 1.25 × 1.25 × 2 mm^3^ voxels, 40 slices, FOV = 120 × 120 mm), and Scan Protocol #2 acquired a reverse EPI sequence.

### 2.7 Macaque MRI preprocessing

MRI preprocessing steps are detailed in prior publications from the research group (Ramirez, 2019; Ramirez et al., 2020; Xu et al., 2018, 2019) and follow the standards for human data and the Adolescent Brain Cognitive Development Study (ABCD Study) project (Ramirez, 2019). Briefly, preprocessing was accomplished by modifying the Human Connectome Project (HCP) minimal preprocessing pipeline (Glasser et al., 2013) for use in macaques (Autio et al., 2020; Donahue et al., 2016). This study utilized version 0.0.0 of the resulting preprocessing pipeline, dcan‐macaque‐pipeline (Center for Developmental NeuroImaging (CDNI), 2025a), and version 0.0.0 of the associated Brain Imaging Data Structure (BIDS) App used to run the pipeline, nhp‐abcd‐bids‐pipeline (Center for Developmental NeuroImaging (CDNI), 2025b), which is also available in newer releases directly on Docker Hub (DCAN Labs, 2025). Study‐specific templates were created from averaged T1w images for each age group (Scott et al., 2016). During structural preprocessing, the age‐matched study‐specific template was registered and warped to each subject's averaged T1w image using tools from the FMRIB Software Library (FSL) (Andersson et al., 2003; Jenkinson et al., 2012; Smith et al., 2004; Woolrich et al., 2009) and Advanced Normalization Tools (ANTs) packages (version 1.9) (Avants, 2025). White and gray matter structures, as well as subcortical regions, were segmented after applying the affine transformations and warps from this registration to the template mask. Segmented and masked structural images were then processed through modified versions of the PreFreeSurfer, FreeSurfer, and PostFreeSurfer stages of the modified HCP pipeline using the FreeSurfer image analysis suite (Dale et al., 1999; Fischl et al., 1999; FreeSurfer, 2025). Subjects were aligned to the Yerkes19 macaque surface‐based atlas (Donahue et al., 2016) for normalized registrations. Functional preprocessing was accomplished in the fMRIVolume and fMRISurface stages of the modified HCP pipeline. These stages included distortion corrections, motion correction, alignments of functional EPI to structural T1w data, resampling, smoothing, and mapping the volumetric data to the standard Connectivity Informatics Technology Initiative (CIFTI) grayordinates space. This grayordinates space consisted of the 56,522 surface anchor points of the Yerkes19 standard space. The final output from this pipeline was a single matrix that contained the cortical surface timeseries and subcortical volume timeseries.

After the MRI data had been processed through the pipeline, trained raters performed a rigorous quality control assessment on the outputs. Raters were blinded to all animal group and demographic information except for the age group and scan protocol. Raters evaluated the data on a scale of 1 to 3, with 1 indicating good quality and 3 indicating poor quality. A score of 2 required a second trained rater to assess the image; the two raters conferred and agreed on whether to include or exclude the subject. Artifacts that merited a subject for exclusion from the study included poor delineations of white and gray matter, brain warping, excessive blurriness, ringing artifacts from motion in the scanner, and in the case of functional data, poor registration to the T1w image or signal dropout over a large area.

Motion censoring was conducted by measuring the total framewise displacement (FD), calculated as the sum of the absolute values of the backward difference for all translation and rotation measures, across a 30 mm brain radius. Frames with FD >0.3 mm were excluded, as well as any isolated frames that came from a group of fewer than five contiguous frames below this threshold, and exactly 20 min of data were extracted from the remainder by selecting frames for inclusion at random. Prior studies have validated this motion correction protocol (Fair et al., 2012; Power et al., 2012, 2014).

Once the functional timeseries data had been quality checked and motion censored, the Bezgin Regional Map parcellation (Bezgin et al., 2012) was applied to assign labeled, monkey‐specific, brain regions of interest (ROIs) to the data. This yielded 82 ROIs belonging to seven functional networks defined in monkeys (Grayson et al., 2016). The correlation between the timeseries signals for every pair of ROIs was calculated using the Pearson product–moment coefficient and saved as a functional connectivity matrix of correlation coefficients (Feczko et al., 2018). The subset of correlation coefficients selected for this analysis consisted exclusively of the correlation coefficients for any pair of ROIs that belonged to either the Auditory, Somatomotor, or Visual networks; the correlation coefficients for any pair that included an ROI from the aforementioned networks and one of the two ROIs that represented the amygdala (one for each brain hemisphere); and the single correlation coefficient denoting the connectivity strength between the pair of amygdala ROIs. In total, this resulted in correlation coefficients for 378 functional connections within and between all of the sensory networks and the amygdala. Specifically, this included connections within and between the 4 ROIs of the Auditory network, the 14 ROIs of the Somatomotor network, the 8 ROIs of the Visual network, and the 2 ROIs from the Limbic network that denote the left‐ and right‐hemisphere amygdala (Table S2).

### 2.8 Statistical analysis of macaque data

The Functional Random Forest (FRF) (Center for Developmental NeuroImaging (CDNI), 2025c) is a machine learning algorithm that constructs a series of decision trees to predict an outcome and has been used by several groups to identify cognitive subtypes within the heterogeneous ASD population from functional connectivity inputs (Cordova et al., 2020; Feczko et al., 2018). For this classification analysis, the input predictors were the 378 correlation coefficients for the set of sensory and amygdala connections, and the outcome measure was the perinatal diet group of the juvenile subject. The model constructed a series of 1000 decision trees. Each tree was trained on a random selection of 90% of the data and tested classification prediction on the remaining 10% of the data. During training, a bootstrapped dataset was randomly selected from a subset of the training data for each tree. A random subset of input features was evaluated at each node where a tree would split; the feature that provided the greatest reduction in classification error was retained in the constructed decision tree. The maximum variable importance, or greatest amount of error reduction, provided by each of the 378 features across model repetitions was exported to identify the features that contributed the most to perinatal diet group distinction. During testing, a random subset of testing data was used to evaluate the accuracy of the model. This was accomplished through 6 repetitions of 5‐fold cross‐validation. The perinatal diet group for each test subject was predicted by each of the 1000 trees in the model, and the final classification was determined by majority vote from the 1000 trees. In addition to predicting correctly labeled data, the model was also used to predict null data, wherein the diet group label had been randomly permuted across subjects. Distributions of overall accuracy, specificity (accurate identification of true negatives when classifying perinatal CTR subjects), and sensitivity (accurate identification of true positives when classifying perinatal WSD subjects) were constructed from the predictions of the observed and null models. A Wilcoxon rank sum test was used to evaluate the significance of these three performance metrics. If all three metrics were significantly better for the observed models than for the null models, then the FRF model was considered valid for predicting perinatal diet group from the set of connectivity features. Further details regarding the implementation of the FRF have been previously described (Cordova et al., 2020; Feczko et al., 2018; Feczko et al., 2019).

For valid FRF models, the variable importance of the features is evaluated to identify the connections that drove accurate model prediction. To assess how these features differed between diet groups, boxplots were generated to inform visual trends; however, further statistical testing for significant differences in the feature distributions was not performed as the features were already identified by a statistically significant analytical model. The most important features were further grouped by the networks to which the component ROIs belonged; intra‐ and inter‐network representation was evaluated to characterize the types of connections that were most important to model performance.

The FRF model was built to predict one outcome variable, so additional factors were not included as covariates. Instead, separate FRF models were constructed to determine whether the same set of connections could be used to predict alternative factors. These models controlled for the impacts of sex and offspring weight at 4 months of age. It was particularly important to test whether connectivity differences could be attributed to offspring sex because the CTR and WSD groups were 73% female and 82% male, respectively. This model was not valid, however, as the Wilcoxon rank sum test did not identify significant differences between the observed and null data across all three performance metrics (Table 2). Offspring weight was evaluated using two FRF models. The first was a classification model that treated weight as a categorical variable with two groups: offspring that weighed <1475 g (*N* = 17), and offspring that weighed more (*N* = 19). The second was a regression model that treated weight as a continuous variable. Model performance for regression was determined by improvements to three measures: the mean absolute error (MAE) in predicting the weight, the correlation between observed and predicted weights (*R*), and the intraclass‐correlation coefficient (ICC). A Student's *t*‐test was used to evaluate the significance of these three performance metrics. Neither of the weight models demonstrated significant predictive capabilities (Table 2). The results of these three models indicate that the connectivity effects associated with perinatal diet group at 4 months of age cannot be attributed to sex or weight.

**TABLE 2:** Performance metrics for models predicting sex and weight from connectivity at 4 months of age.

Classification	Overall accuracy		Specificity		Sensitivity	
	Observed	Permuted	Observed	Permuted	Observed	Permuted
Sex						
Mean	53.8	43.6	60.8	50.3	46.7	41.7
SD	17.8	18.1	24.3	29.2	25.1	29.4
*p* value	0.038		0.147		0.573	
*Z* value	−2.08		−1.45		−0.56	
Weight						
Mean	45.2	41.5	31.9	34.9	58.1	53.7
SD	20.2	15.6	29.6	25.6	29.1	24.6
*p* value	0.538		0.642		0.441	
*Z* value	−0.62		−0.47		−0.77	

Regression	MAE		*R*		ICC	
	Observed	Permuted	Observed	Permuted	Observed	Permuted
Weight						
Mean	113.4869	118.1162	0.0706	0.0362	0.9069	0.9045
SD	25.9788	42.2387	0.2899	0.4551	0.0089	0.0164
*t*‐statistic	−0.51		0.35		0.72	
df	58		58		58	
*p* value	0.611		0.728		0.475	
95% CI	[−22.75, 13.493]		[−0.163, 0.23]		[−0.004, 0.009]	

*Note*: Performance metrics are presented for the functional random forest (FRF) classification and regression models of observed and permuted data predicting either offspring sex or weight from the functional connectivity of 378 sensory and amygdala connections at 4 months of age. Means for the classification metrics are percentages referring to the classification accuracy. Significance was determined by a Wilcoxon rank sum test for classification models and a Student's *t*‐test for the regression model.

Abbreviations: CI, confidence interval; df, degrees of freedom; ICC, intraclass‐correlation coefficient; MAE, mean absolute error; *R*, Pearson's correlation coefficient; SD, standard deviation.

## 3 RESULTS

### 3.1 Sensory connections accurately predicted perinatal diet exposure in macaques at 4 months of age

**FIGURE 2:**
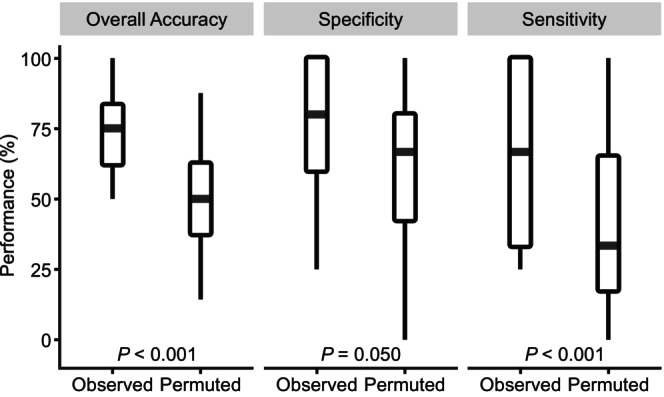
Performance metrics for the functional random forest (FRF) model in macaques at 4 months of age. The functional connectivity of 378 connections within and between all sensory network regions and the amygdala was used to predict control (CTR) or Western‐style diet (WSD) perinatal diet exposure. Distributions of overall accuracy, specificity (accurate identification of true negatives when classifying perinatal CTR subjects), and sensitivity (accurate identification of true positives when classifying perinatal WSD subjects) were constructed from the predictions of the observed and permuted models. Statistical significance across all three metrics was required for a model to be considered valid for predicting perinatal diet group. A valid model was achieved at 4 months of age (*N* = 39, CTR *n* = 22, female *n* = 19), with the observed model demonstrating a significant improvement in overall accuracy (*p* < 0.001, *Z* = −4.62), specificity (*p* = 0.050, *Z* = −1.96), and sensitivity (*p* < 0.001, *Z* = −3.60). The central line represents the median. Wide bars refer to the 25th/75th percentiles; thinner bars refer to the 2.5th/97.5th percentiles. Significance was determined by a Wilcoxon rank sum test.

FRF model performance metrics are shown in Figure 2. The FRF model used 378 functional connectivity features between sensory networks and the amygdala to classify offspring by perinatal diet group with a mean overall accuracy of 73.9% at 4 months of age. A Wilcoxon rank sum test revealed that this model was significantly more accurate than the permuted model which achieved 51.1% overall accuracy (*p* < 0.001, *Z* = −4.62). The specificity and sensitivity of the model at the 4‐month‐old time point were also significantly greater than those metrics for the permuted models. The model had a specificity of 78.3% when classifying offspring exposed to a perinatal CTR diet, indicating the ability of the model to correctly identify true negatives, and a Wilcoxon rank sum test determined that this was significantly more accurate than the permutation specificity of 64.1% (*p* = 0.050, *Z* = −1.96). The model had a sensitivity of 66.9% when classifying offspring exposed to a perinatal WSD, indicating the ability of the model to correctly identify true positives, and a Wilcoxon rank sum test determined that this was significantly more accurate than the permutation sensitivity of 37.6% (*p* < 0.001, *Z* = −3.60). Given that these three metrics were significantly improved in the model with correctly labeled, observed data, this indicates that the FRF model is valid for predicting perinatal diet group from the set of connectivity features. Perinatal exposure to a WSD alters the set of sensory and amygdala connectivity strengths at 4 months of age to the extent that they can be used to predict the perinatal diet exposure for any given subject.

Several supplemental and preliminary analyses were pursued across offspring development at 6, 11, 21, and 36 months of age. A separate FRF model was created for each age group in a longitudinal analysis, but no model achieved significance across all three performance metrics (Figure S1). Thus, the models are not valid for predicting perinatal diet exposure at the later developmental time points. The functional connectivity features from the 36‐month age group were additionally used to predict two other measures: (1) another proposed prenatal predictor of SPD, the pre‐pregnancy adiposity of each subject's mother (Table S3, Figure S2), and (2) a potential mediator of altered connectivity, the number of microglia and macrophages in the amygdala of each subject (Table S4, Figures S3 and S4). Consistent with the perinatal diet model, neither of these measures was predictive of connectivity at 36 months. Finally, this study explored the functional connectivity patterns of children that exhibited a measure of SPD. Three FRF models were run on slightly different subsets of human imaging data, but none of the models demonstrated significant predictive capabilities (Table S5, Figure S5). Thus, these models were not valid for predicting a child's SOR score from the functional connectivity within and between their sensory networks and the amygdala.

### 3.2 Intra‐somatomotor and visual‐auditory network connections disproportionately aided model prediction

The FRF model accurately classified subjects at 4 months of age based on 378 functional connectivity input features. Features that differed more between groups likely played a more prominent role in helping the decision tree distinguish between groups. One way to measure the importance of a feature to a model is by calculating the variable importance, or the sum of the decrease in error when a decision tree is split by that feature. The variable importance of each of the 378 features was calculated for each of the six model repetitions that were run. The maximum variable importance that each feature achieved across the six repetitions was selected.

**FIGURE 3:**
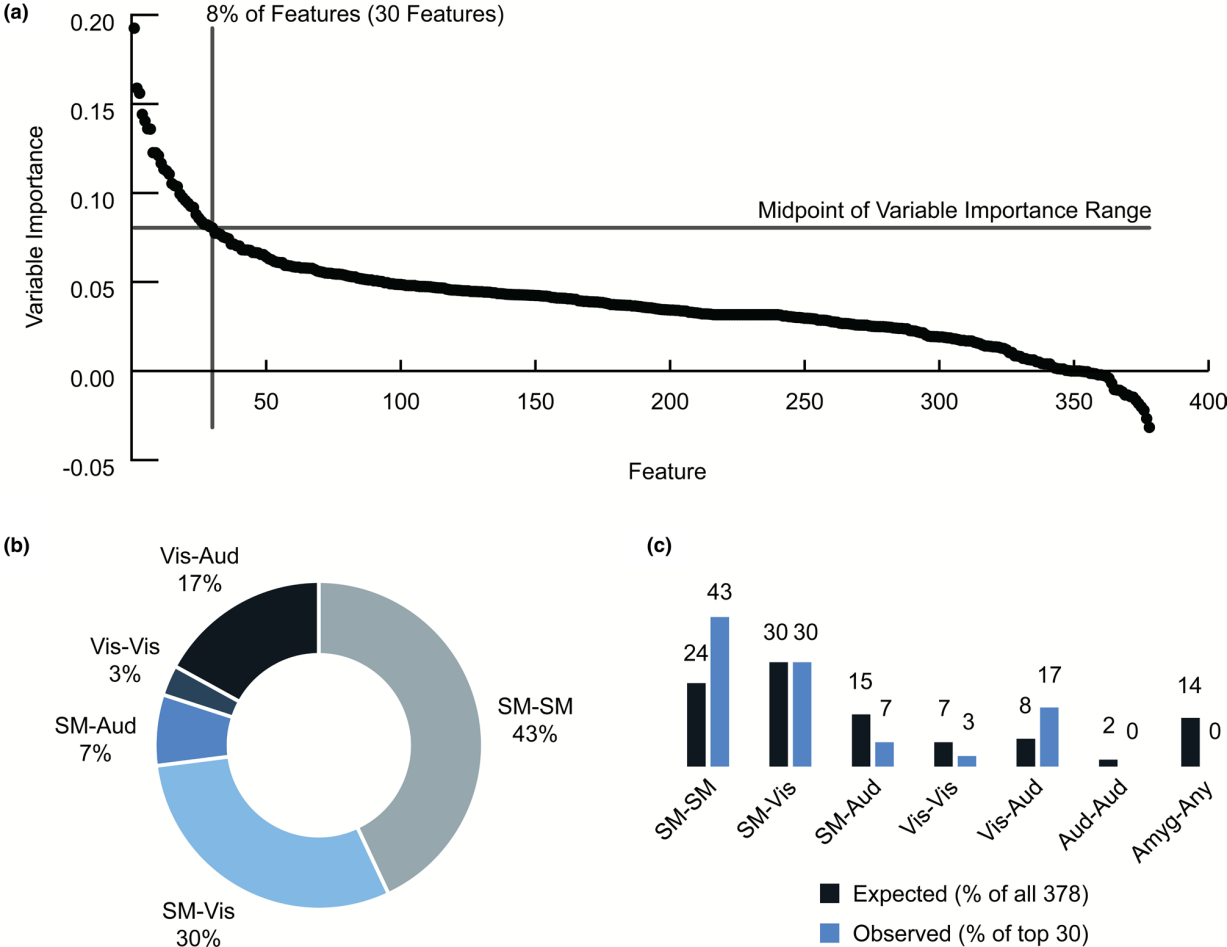
Top 30 features driving performance of the functional random forest (FRF) model of macaque connectivity at 4 months of age. The model was trained on functional connectivity features from 39 offspring at 4 months of age (CTR *n* = 22, female *n* = 19) and validated for predicting perinatal diet group. (a) The maximum variable importance value for each of the 378 functional connectivity features is displayed. The 378 features are ordered along the *x*‐axis from highest to lowest maximum variable importance value. The midpoint of the range of variable importance values is marked with a horizontal line at 0.080. A vertical line at position 30 along the *x*‐axis visually separates the 30 features that achieved a maximum variable importance value greater than the midpoint of the range of values from the remaining features. (b) Chart displaying the percentage of the 30 most important features that belong to each of the represented network groupings. (c) Comparison of network grouping representation. Dark blue bars, on the left side of each pair, display the percentage of all 378 features that belong to the respective network grouping. Lighter blue bars, on the right side of each pair, display the percentage of the 30 most important features that belong to the respective network grouping. Abbreviations from b and c: Amyg‐Any, amygdala‐any network; Aud‐Aud, auditory‐auditory; SM‐Aud, somatomotor‐auditory; SM‐SM, somatomotor‐somatomotor; SM‐Vis, somatomotor‐visual; Vis‐Aud, visual‐auditory; Vis‐Vis, visual‐visual.

The maximum variable importance values for the 378 features ranged from −0.032 to 0.193 (Figure 3a), where a more positive value denoted a greater reduction in error and, therefore, a more important feature. The midpoint of this range is 0.080; thus, a maximum variable importance value of 0.080 or above denoted an importance to the model that was greater than at least half of all possible importance values. Only 8% of features, or a total of 30 features, achieved a maximum variable importance value that was in the upper half of the demonstrated range.

Each feature represents a functional connection between two ROIs. Each ROI belongs to a functional network. The 30 most important features were grouped by the networks represented by each ROI in the connection pair. For example, the most important feature with a maximum variable importance of 0.193 belonged to the functional connection between the primary motor cortex ROI of the left hemisphere and the medial premotor cortex ROI of the left hemisphere. Each ROI was a member of the somatomotor network, so the network grouping for this connection would be the “somatomotor‐somatomotor” or “intra‐somatomotor” group. For clarity, any grouping that included a connection to the amygdala contained the term “amygdala” in the grouping name rather than its functional network, “limbic.”

The 30 most important features belonged to five network groups, with the greatest proportion of features, at 43%, deriving from the somatomotor‐somatomotor network group (Figure 3b). Another 30% of features were connections between the somatomotor and visual networks, 17% were visual‐auditory connections, 7% were somatomotor‐auditory connections, and 3% were visual‐visual connections.

These proportions were not representative of the overall distribution of network groupings across the full set of 378 connections (Figure 3c). For example, somatomotor‐somatomotor network connections comprised just 24% of all 378 connections, yet they comprised 43% of the 30 most important features, indicating that they were over‐represented in the set of features that drove model performance. Visual‐auditory connections were also over‐represented, encompassing 17% of the 30 most important features when only an 8% representation was expected by chance. On the other hand, the somatomotor‐auditory group, visual‐visual group, and the combined groups of features that contained any connection to the amygdala were all underrepresented in the set of 30 most important features. Two groups, the somatomotor‐visual and auditory–auditory features, were included in the set of 30 most important features in proportions that were representative of their respective shares among all 378 features.

The 30 most important features are listed in Table S6. Notably, many individual ROIs appear more than once. Table S7 summarizes the ROIs that appeared in at least three of the 30 most important features. The right primary motor cortex and left primary somatosensory cortex were the two connections that appeared the most, appearing in 7 and 5 features respectively. Both of these ROIs are part of the somatomotor network, demonstrating again that differences in somatomotor network connectivity were strong drivers of model performance.

### 3.3 Perinatal WSD exposure was associated with consistent, network‐specific changes in connectivity strength

**FIGURE 4:**
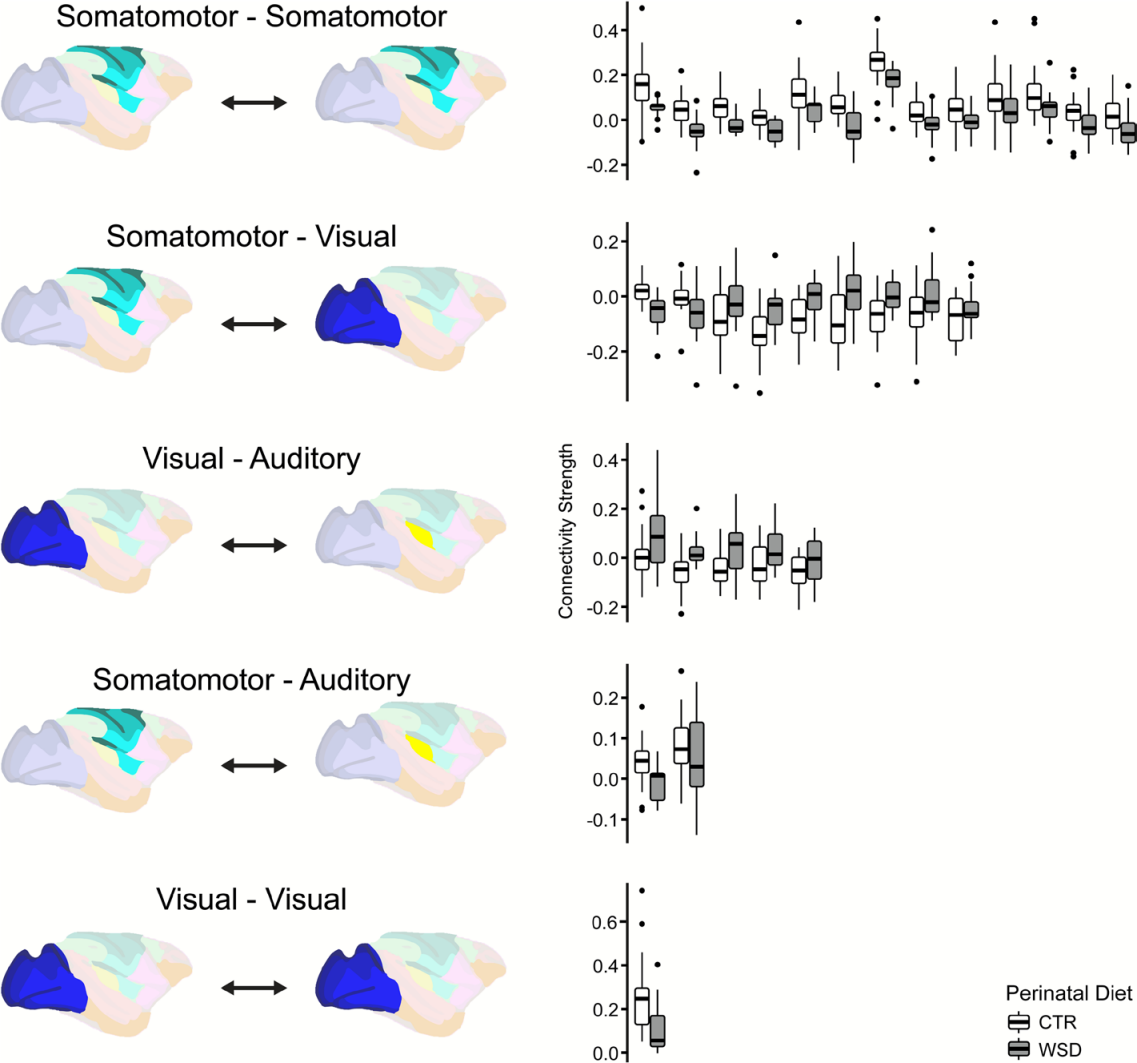
Distributions of connectivity strengths between perinatal diet groups for the 30 features with the greatest maximum variable importance from the macaque functional random forest (FRF) model at 4 months of age. Boxplot pairs, each representing one of the 30 features, are clustered by network group and are ordered along the *x*‐axis from highest to lowest maximum variable importance value. White boxplots, on the left side of each pair, represent the distribution for the perinatal control diet (CTR) offspring. Gray boxplots, on the right side of each pair, represent the distribution for the perinatal Western‐style diet (WSD) offspring. The central line represents the median. Wide bars refer to the 25th/75th percentiles; thinner bars refer to the 2.5th/97.5th percentiles. Significance testing was not performed on these distributions as they were already identified as being important for a statistically significant model prediction. The model included data from 39 offspring at 4 months of age (CTR *n* = 22, female *n* = 19).

Each of the 30 features that achieved the greatest maximum variable importance in the FRF model constructed on the 4‐month‐old offspring data was assessed for differences between perinatal diet groups. The distribution of connectivity strengths, measured as correlation coefficients, among offspring exposed to a perinatal WSD and CTR was compared for each feature (Figure 4). For example, the feature that contributed the most to accurate model performance was the connection between the left primary motor cortex and left medial premotor cortex. Offspring that were perinatally exposed to a WSD showed decreased connectivity in that feature at 4 months of age, with a mean connectivity strength of 0.057 (SD 0.041), compared to controls, with a mean of 0.158 (SD 0.139).

All of the features that belonged to the two network groupings that were over‐represented in the 30 most important features followed a consistent pattern of altered connectivity (Figure 4). The perinatal WSD offspring displayed weaker connectivity across all 13 connections in the somatomotor‐somatomotor network group, and they displayed stronger connectivity across all 5 connections in the visual‐auditory network group. The two connections in the somatomotor‐auditory network group were also consistent, with decreased connectivity in the perinatal WSD group. Differences in connectivity strength were mixed for the somatomotor‐visual network group. While perinatal WSD offspring displayed trends toward decreased connectivity for the first two most important connections in the network group, the remaining seven connections were characterized by trends toward increased connectivity. The only connection for the visual‐visual network group demonstrated decreased connectivity for the perinatal WSD group. Significance testing was not performed on these distributions as they were already identified as being important for a statistically significant model prediction.

### 3.4 Amygdala‐somatomotor connectivity weakly contributed to model performance

Connections to the amygdala were not present in the 30 most important features. However, amygdala connectivity may still be a weak driver of accurate model performance. While amygdala connections were not present in the 30 most important features, or the top 8% of all 378 features, four amygdala connections were present in the top 15% of all features, or within the 56 most important features. These four connections were between the amygdala and somatomotor network (Table S8), and they ranked at 44, 47, 49, and 50 when all features were ordered by maximum variable importance. The maximum variable importance values for these connections ranged from 0.068 to 0.064 (highest to lowest). A maximum variable importance of 0.064 is >46% of the values in the range exhibited by the model. This suggests that amygdala‐somatomotor connectivity may still provide valuable information to the model as it learns to distinguish between diet groups.

**FIGURE 5:**
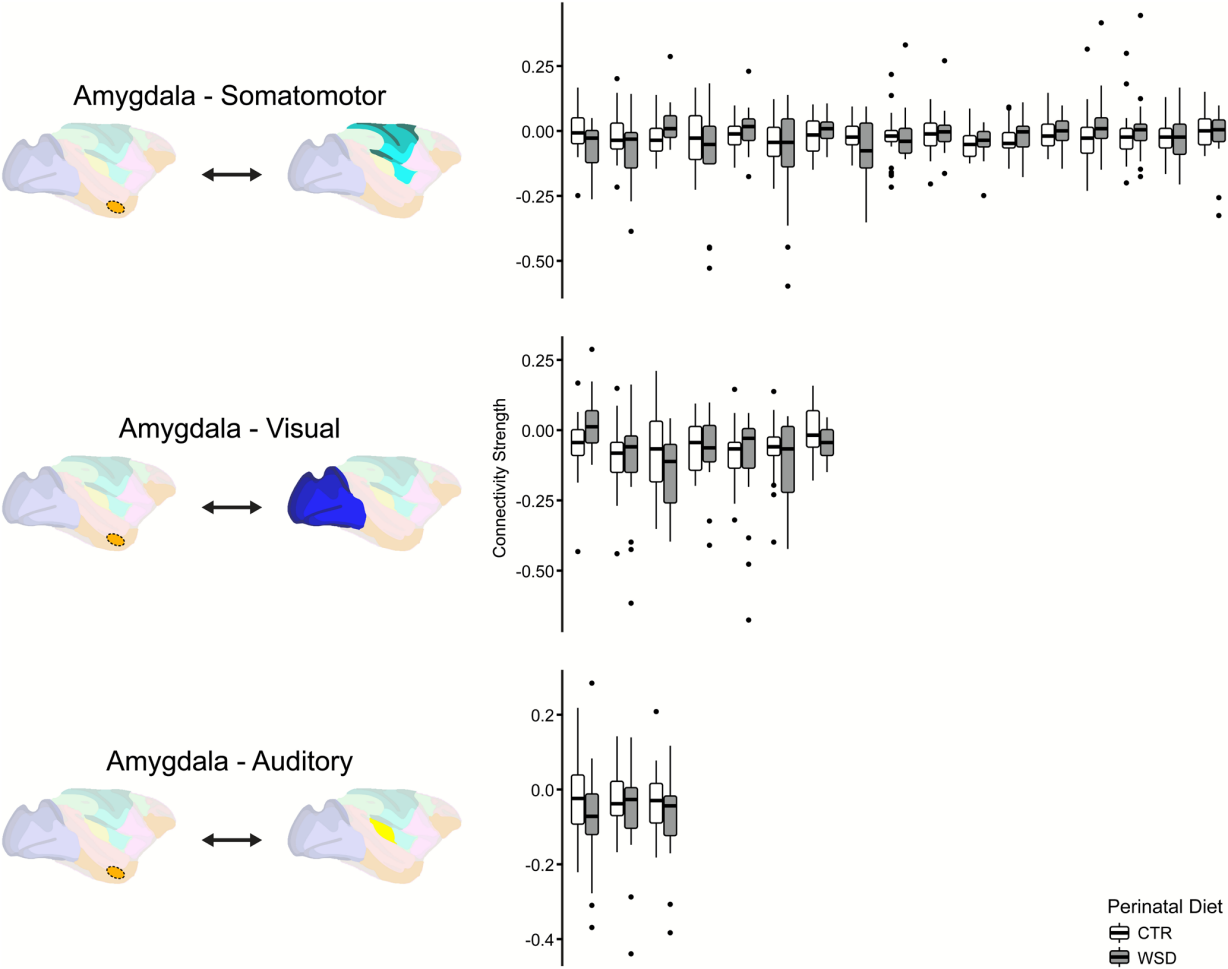
Distributions of connectivity strengths between perinatal diet groups for amygdala connections within the upper 50% of features with the greatest maximum variable importance from the macaque functional random forest (FRF) model at 4 months of age. Boxplot pairs, each representing a functional connection with the amygdala, are clustered by network group and are ordered along the *x*‐axis from highest to lowest maximum variable importance value. White boxplots, on the left side of each pair, represent the distribution for the perinatal control diet (CTR) offspring. Gray boxplots, on the right side of each pair, represent the distribution for the perinatal Western‐style diet (WSD) offspring. The central line represents the median. Wide bars refer to the 25th/75th percentiles; thinner bars refer to the 2.5th/97.5th percentiles. Significance testing was not performed on these distributions as they were already identified as being important for a statistically significant model prediction. The model included data from 39 offspring at 4 months of age (CTR *n* = 22, female *n* = 19).

The distributions of amygdala connectivity strengths were characterized by weak differences between diet groups (Figure 5). Additionally, when looking across the amygdala connections that were present within the upper 50% of most important features (or the 189 most important features), a consistent pattern of altered connectivity did not emerge in any network grouping. Thus, amygdala‐somatomotor connectivity, while constituting the most important of the amygdala connections, may have only weakly contributed to accurate model performance in macaques at 4 months of age.

## 4 DISCUSSION

There are several conflicting theories about the origins of the symptoms of SPD. While some evidence suggests SPD may be a manifestation of other root psychiatric conditions, other data support the hypothesis that SPD may stem from direct disruptions to sensory processing, in some cases leading to the emotional dysregulation seen in psychiatric conditions as a consequence of the experience of living with the symptoms of SPD. This study took the position of the latter theory and explored the connectivity of a subset of brain regions that represent the primary cortical areas for processing sensory input. Prior evidence suggested that connections to the amygdala may be a strong component of ascending sensory processing pathways, especially in non‐classical sensory pathways that may persist in adults with ASD (Møller et al., 2005; Moller & Rollins, 2002; Musiek et al., 2011). For this reason, the amygdala was included in the ROI subset, as well. The amygdala is intrinsic to the processing of negative emotions, so even if purely‐sensory connectivity was not found to be disrupted, alterations to amygdala connectivity could support the hypothesis that emotional dysregulation is a driving factor behind the symptoms of SPD.

While the juvenile macaques in this study were not tested explicitly for symptoms of SPD, prior work in this model established that the offspring who were exposed to a WSD during the perinatal period displayed the component behaviors of ASD, including sensorimotor behaviors at 6.6 months of age that involved abnormal movements, posture, and increased head tossing and pacing (Mitchell et al., 2022). Given that an estimated 95% of children with ASD experience a SPD (Crane et al., 2009; Leekam et al., 2007; Tomchek & Dunn, 2007), it was expected that the perinatal WSD group would also exhibit altered circuitry in sensory or emotional processing areas.

This expectation was supported when offspring were 4 months old. A follow‐up characterization of the 4 month model results revealed that differences in intra‐somatomotor, visual–auditory, somatomotor‐auditory, somatomotor‐visual, and intra‐visual network connections were the most distinct between perinatal diet groups, with the first two network groupings being over‐represented in the set of the 30 features that contributed the most to improving model prediction. Connectivity was impacted in the same direction within each network grouping except the somatomotor‐visual group, demonstrating a largely consistent impact of perinatal WSD exposure on network‐wide connectivity. Impacts to the connectivity of the primary motor cortex seemed to be the most pronounced, as the ROI was part of the connection that was identified as being the most important to the model, and it appeared in seven of the 30 most important connections, more than any other ROI. The preponderance of somatomotor impacts may explain the sensorimotor behaviors observed in the WSD group (Mitchell et al., 2022). Amygdala connectivity was not found to be a strong contributor to model performance, though weak differences in connectivity may have still improved the model. Amygdala‐somatomotor connections, in an inconsistent pattern within the network grouping, were within the top 15% of connections when ordered by importance. Amygdala connections to the significantly impacted somatomotor network could explain the emotional component of SOR, which is predominantly experienced solely in the tactile domain (Ben‐Sasson et al., 2009).

Although the same set of sensory and amygdala connections were unable to predict a measure of SOR in a cohort of 9 and 10‐year‐old children, this finding is consistent with the poor model performance in macaques at the same developmental time point at 36 months of age. Additionally, supplemental analyses were unable to use connectivity from the 36‐month‐old macaques to predict maternal adiposity, which is a related potential predictor of SPD, and the number of microglia in the amygdala, which can increase in response to neuroinflammation and can serve as a mediator for altered connectivity (Belhocine et al., 2022; Cowan & Petri Jr., 2018; Huang et al., 2016; Kim et al., 2017; Kleinhans et al., 2016; Lively & Schlichter, 2013; Sarlus & Heneka, 2017; Ypma et al., 2016). These null results suggest that the connectivity patterns at this age are relatively robust against influences associated with perinatal WSD exposure, though additional studies with larger sample sizes and more sensitive analytical techniques are needed to confirm this general trend.

### 4.1 Functional connectivity impacts at early life stages may arise from WSD‐associated maternal inflammation during a period of rapid synaptogenesis

Neurodevelopment proceeds through different stages and may be more susceptible to disruptions at different times. The third trimester in particular is a highly dynamic period characterized by rapid cortical maturation and dramatically increased neuronal connectivity (Andescavage et al., 2017; Tau & Peterson, 2010; Thomason et al., 2015; van den Heuvel et al., 2015). Rapid synaptogenesis continues through the first year of life, and synaptic pruning does not outpace synapse formation until later in childhood (Eltokhi et al., 2020; Levitt, 2003). Disruptions during the third trimester and first year of life, when cerebral connectivity is undergoing its greatest period of development, may therefore be more pronounced than effects that occur at other time points.

When comparing developmental stages across species, a 4‐month‐old rhesus macaque is roughly the equivalent of an 8‐month‐old human (Charvet et al., 2022; Workman et al., 2013), which falls within the first year of comparable human development in which connections are undergoing dramatic construction. Neuroinflammation could therefore pose a significant disruption to microglia‐mediated circuit formation during this time. It is well established that WSD consumption during pregnancy can lead to an elevated inflammatory state, and that increased maternal inflammation can lead to increased fetal inflammation (Grayson et al., 2010). In fact, prior work in this model has demonstrated an elevated neuroinflammatory state in the fetal hypothalamus with exposure to a prenatal WSD, though follow‐up studies at 11 months of age revealed a slight decrease in the number of microglia in the hypothalamus and amygdala of the WSD offspring, suggesting that the neuroinflammatory state recovers and is even reduced compared to controls <1 year after birth (Dunn, Mitchell, et al., 2022; Grayson et al., 2010; Papadakis et al., 2024). Given that 11 months in rhesus macaques is the equivalent of 36 months in humans (Charvet et al., 2022; Workman et al., 2013), this finding coincides with a developmental time point beyond the period of dynamic circuit formation. The timing of an earlier period of heightened neuroinflammation suggests that the microglial process of neural precursor cell phagocytosis, which is prominent during the third trimester, may be responsible for the altered circuitry seen at 4 months. Increased neuroinflammation and microglial prevalence are associated with increased phagocytosis of neural precursor cells (Cunningham et al., 2013), so it is possible that the number of neurons that reached maturity was reduced in areas that demonstrated decreased connectivity at 4 months.

Although the proposed mechanisms are speculative in the context of the present study's limited scope, these findings suggest that functional connectivity between the sensory systems and the amygdala may be susceptible to perturbations deriving from the inflammatory prenatal environment during the coinciding period of dynamic circuit formation. Future research should investigate the mechanisms that contribute to altered neuronal structuring in the specific set of sensory circuits that are impacted during early development, and determine whether these circuits are retained or modified during later periods of reduced neuroinflammation.

### 4.2 Functional connectivity outcomes are consistent between macaque and human cohorts

A recent study has identified differences in discrete connections using the same human cohort as in the supplemental analysis of the present study (Schwarzlose et al., 2023). This study tested many network groupings, though a significant difference was found for only one network grouping within the set examined in the present study: decreased intra‐somatomotor network connectivity in children with SOR.

The isolated effect of decreased intra‐somatomotor connectivity resembles the results from the 4‐month‐old macaque data which found decreased intra‐somatomotor connectivity to be the strongest driver of model performance. The consistent direction of decreased connectivity in the perinatal WSD and highly symptomatic SOR groups hints at a link between the dietary exposure and the behavioral outcome. It also suggests a plausible trajectory where the strongest differences at 4 months of age may persist and present as some of the only remaining differences in sensory connectivity at a later age. Altered intra‐somatomotor connectivity is also consistent with behavioral findings that show that most children with SOR experience over‐responsivity exclusively in the somatosensory modality (Ben‐Sasson et al., 2009).

Taken together, the human studies provide outcomes that are consistent with the results demonstrated in the macaque analyses, encouraging further investigation into the relationship between perinatal WSD exposure and altered sensory connectivity.

## 5 LIMITATIONS

The FRF is a powerful tool for predicting an outcome measure from a large set of independent and dependent features, but there are limitations to its capabilities. A primary limitation is the reliance on widespread differences across many features between groups, or at least dramatic differences if restricted to a smaller subset of features. The FRF is not as effective at identifying differences in isolated features, as it is designed to learn patterns associated with each group rather than test for significant differences for each feature. Analyses that implement other approaches could be useful in identifying more subtle or focal differences in connectivity associated with perinatal WSD exposure.

Another major limitation of this study was the small sample sizes for the macaque data. Increased sample sizes may have reduced the variability in the connectivity patterns observed within each age group in the supplemental longitudinal analysis, which would have aided FRF model prediction.

More sensitive techniques to analyze highly variable brain connectivity, combined with larger, balanced sample sizes, should enable future studies to assess longitudinal impacts in brain circuitry in the context of SPD. Additionally, a cross‐species comparison of functional connectivity in 8‐month‐old humans with SOR symptoms—or who later develop SOR symptoms—to the 4‐month‐old macaques in this study could test for a relationship between perinatal WSD exposure and SOR, increasing what is known about perinatal predictors of SPD.

## 6 CONCLUSIONS

Perinatal WSD exposure was associated with altered connectivity in sensory and emotional processing areas at 4 months of age. These impacts are characterized primarily by decreased connectivity within the somatomotor network, within the visual network, and between the somatomotor and auditory networks; increased connectivity between the auditory and visual networks; and mixed effects between the somatomotor and visual networks. The prominence of somatomotor impacts corresponds to human SPD studies that similarly demonstrate decreased intra‐somatomotor connectivity and predominantly somatomotor‐based SOR behavioral symptoms. Connections to the amygdala were only weakly impacted. These results suggest that a prenatal predictor of behaviors characteristic of ASD, a disorder that is highly comorbid with SPD, may disrupt sensory connectivity during infancy, supporting the theory that altered sensory processing may serve as an origin for the symptoms of SPD. The period of rapid synaptogenesis during the third trimester and first few months of life may be particularly susceptible to the impacts of WSD exposure, which include a strong inflammatory response in utero. Inflammatory disruptions to microglia‐mediated circuit formation during this period could explain the differences in sensory connectivity that are still present at 4 months. Taken together, these results suggest that the functional connectivity of sensory networks and the amygdala are impacted by perinatal WSD exposure consistent with patterns seen in SPD. However, the nature of the FRF analysis and the limitations of small sample sizes mean that nuanced differences in these or other sensory connections cannot be ruled out. Follow‐up analyses could probe for focal differences in specific connections to ascertain whether any sensory‐specific impacts remain at later time points. Further research is needed to understand the remodeling mechanisms that alter brain circuitry by 4 months of age and the processes that may mitigate differences at later ages. Lastly, additional cross‐species comparisons are encouraged to better determine whether perinatal WSD exposure is a prenatal predictor of SPD.

## DISCLAIMERS

The funders played no role in the writing of this content or in the decision to submit the article for publication. None of the data has been previously published.

## AUTHOR CONTRIBUTIONS

Samantha Papadakis, Elinor L. Sullivan, and Damien A. Fair conceived and designed research; Samantha Papadakis, Julian S. B. Ramirez, AJ Mitchell, and Geoffrey A. Dunn performed experiments or MRI preprocessing; Samantha Papadakis analyzed data; Samantha Papadakis, Eric Feczko, Oscar Miranda‐Dominguez, and Damien A. Fair interpreted results of experiments; Samantha Papadakis prepared figures. Samantha Papadakis drafted manuscript. Samantha Papadakis, Eric Feczko, Oscar Miranda‐Dominguez, Eric Earl, Elinor L. Sullivan, and Damien A. Fair edited and revised manuscript; Samantha Papadakis, Eric Feczko, Julian S. B. Ramirez, Oscar Miranda‐Dominguez, Darrick Sturgeon, Thomas J. Madison, Anders J. Perrone, Eric Earl, AJ Mitchell, Geoffrey A. Dunn, Elinor L. Sullivan, and Damien A. Fair approved final version of manuscript.

## FUNDING INFORMATION

This work was funded by the National Institutes of Health under grant number R01MH107508 to Elinor L. Sullivan and grant numbers 2R01MH096773‐08A1, 5R01MH096773‐09, and U01DA041148 to Damien A. Fair, as well as grant number P51‐OD011092 for the operation of ONPRC and support of the Magnetic Resonance Imaging Core.

## CONFLICT OF INTEREST STATEMENT

No conflicts of interest, financial or otherwise, are declared by the authors.

## Supporting information


Data S1.

## Data Availability

Data will be made available upon reasonable request.
