# Supplementary material for: Perinatal Western‐style diet exposure associated with altered sensory functional connectivity in infant Japanese macaques
Source: Physiol Rep. 2025 Dec 2;13(23):e70674. doi: 10.14814/phy2.70674 (PMC12673176; doi:10.14814/phy2.70674)
Supplement: Supplementary file 1 — Data S1. [file PHY2-13-e70674-s001.docx]

**SUPPLEMENTAL MATERIAL FOR:**

RESEARCH ARTICLE

Perinatal Western-style diet exposure associated with altered sensory functional connectivity in infant Japanese macaques

Samantha Papadakis,^1,2^ Eric Feczko,^3,4^ Julian S. B. Ramirez,^1^ Oscar Miranda-Dominguez,^3,4^ Darrick Sturgeon,^1^ Thomas J. Madison,^4^ Anders J. Perrone,^4^ Eric Earl,^2^ AJ Mitchell,^1,5^ Geoffrey A. Dunn,^6^ Elinor L. Sullivan,^1,2,5^ Damien A. Fair^3,4,7^

^1^ Department of Behavioral Neuroscience, Oregon Health & Science University, Portland, Oregon, United States

^2^ Department of Psychiatry, Oregon Health & Science University, Portland, Oregon, United States

^3^ Department of Pediatrics, University of Minnesota Medical School, Minneapolis, Minnesota, United States

^4^ Masonic Institute for the Developing Brain, University of Minnesota Medical School, Minneapolis, Minnesota, United States

^5^ Division of Neuroscience, Oregon National Primate Research Center, Oregon Health & Science University, Beaverton, Oregon, United States

^6^ Department of Human Physiology, University of Oregon, Eugene, Oregon, United States

^7^ Institute of Child Development, College of Education and Human Development, University of Minnesota, Minneapolis, Minnesota, United States

Correspondence: *Damien A. Fair (faird@umn.edu).*

# SUPPLEMENTAL MATERIAL

[Supplemental Table S1 and Fig. S1](#_SUPPLEMENTAL_TABLE_S1): Pages 2-9.

[Supplemental Table S2](#_SUPPLEMENTAL_TABLE_S2): Page 11.

[Supplemental Table S3 and Fig. S2](#_SUPPLEMENTAL_TABLE_S3): Pages 13-15.

[Supplemental Table S4 and Figs. S3 and S4](#_SUPPLEMENTAL_TABLE_S4): Pages 17-21.

[Supplemental Table S5 and Fig. S5](#_SUPPLEMENTAL_TABLE_S5): Pages 23-30.

[Supplemental Tables S6 and S7](#_SUPPLEMENTAL_TABLES_S6): Pages 32-34.

[Supplemental Table S8](#_SUPPLEMENTAL_TABLE_S8): Pages 36-37.

# SUPPLEMENTAL TABLE S1 AND FIG. S1

**MATERIALS AND METHODS**

Animal Model

The supplemental analyses at 6, 11, 21, and 36 months of age used the same animal model as described in the main text for the 4 month old subjects. Differences unique to the supplemental analyses are reported in the affected sections below.

All animal group demographic measures are presented for the full cohort of 69 subjects selected for brain scanning across all time points, including those scanned at 4, 6, 11, 21, and 36 months of age.

All animal procedures were in accordance with National Institutes of Health guidelines on the ethical use of animals and were approved by the Oregon National Primate Research Center (ONPRC) Institutional Animal Care and Use Committee.

Adult Female Macaques

Mean maternal age at offspring birth was 10.81 yr (SD 3.19) for the control diet (CTR) group and 8.52 yr (SD 2.46) for the Western-style diet (WSD) group. Mean maternal pre-pregnancy weight was 9.92 kg (SD 1.82) for the CTR group and 9.96 kg (SD 2.18) for the WSD group, with one missing value for one of the WSD offspring.

Macaque Juvenile Offspring

Offspring were weaned at a mean age of 7.22 mo (SD 1.62) for the CTR group and 7.50 mo (SD 1.14) for the WSD group (7 and 3 missing values for the CTR and WSD groups, respectively). At this time, offspring were relocated to group housing with 6-10 juveniles of a similar age and 1-2 unrelated adult females. This study included maternal siblings (43 of the 69 included subjects had at least one sibling in the study, with all 43 born from a total of 17 dams), though paternal identification was unknown.

A total of 81 juveniles were selected for this study. Subjects were excluded for medical issues, a change in maternal diet partway through gestation, and poor quality scans at every available age point. Of the 69 juveniles who remained in the study, 48 of them contributed acceptable-quality scans at more than one time point, and 37 had quality scans at three or more time points. This study aimed to balance for perinatal diet group and offspring sex (*N* = 69; CTR *n* = 33, female *n* = 21; WSD *n* = 36, female *n* = 13). However, to increase the sample size for use with functional connectivity analyses, two additional criteria were permitted. While the majority of the 69 juvenile subjects consumed the CTR diet after weaning, 14 consumed a post-weaning WSD (Perinatal CTR *n* = 7, female *n* = 4; Perinatal WSD *n* = 7, female *n* = 4). This introduced a dietary difference only for the three older age groups, as the first two scans occurred prior to weaning. Additionally, this study combined scans that were collected under two separate acquisition protocols, as described in the main text. These two factors were not accounted for as a covariates in the classification analyses of this study. While this presents a limitation, prior work in this model has established that post-weaning diet group does not have a significant effect on amygdala volume growth across the time points included in this study (1), and the change in scan acquisition protocol does not have a significant effect on mean cortical thickness across these same time points (2).

**Table S1.** Demographic composition of macaque subjects at each age time point

| **Subjects in 4-month Group** | | | | | | | | |
| --- | --- | --- | --- | --- | --- | --- | --- | --- |
| Scan Protocol | #1 | | | | #2 | | | |
| Perinatal Diet | CTR | | WSD | | CTR | | WSD | |
| Post-weaning Diet | CTR | WSD | CTR | WSD | CTR | WSD | CTR | WSD |
| Male | 3 | 0 | 9 | 0 | 3 | 0 | 5 | 0 |
| Female | 7 | 0 | 2 | 0 | 7 | 2 | 1 | 0 |
| Total | 39 | | | | | | | |
| **Subjects in 6-month Group** | | | | | | | | |
| Scan Protocol | #1 | | | | #2 | | | |
| Perinatal Diet | CTR | | WSD | | CTR | | WSD | |
| Post-weaning Diet | CTR | WSD | CTR | WSD | CTR | WSD | CTR | WSD |
| Male | 1 | 0 | 5 | 0 | 3 | 0 | 4 | 0 |
| Female | 3 | 0 | 0 | 0 | 7 | 2 | 0 | 0 |
| Total | 25 | | | | | | | |
| **Subjects in 11-month Group** | | | | | | | | |
| Scan Protocol | #1 | | | | #2 | | | |
| Perinatal Diet | CTR | | WSD | | CTR | | WSD | |
| Post-weaning Diet | CTR | WSD | CTR | WSD | CTR | WSD | CTR | WSD |
| Male | 1 | 1 | 6 | 2 | 3 | 0 | 7 | 0 |
| Female | 4 | 2 | 1 | 2 | 7 | 1 | 0 | 0 |
| Total | 37 | | | | | | | |
| **Subjects in 21-month Group** | | | | | | | | |
| Scan Protocol | #1 | | | | #2 | | | |
| Perinatal Diet | CTR | | WSD | | CTR | | WSD | |
| Post-weaning Diet | CTR | WSD | CTR | WSD | CTR | WSD | CTR | WSD |
| Male | 1 | 1 | 6 | 2 | 2 | 0 | 6 | 0 |
| Female | 4 | 2 | 3 | 2 | 3 | 0 | 0 | 0 |
| Total | 32 | | | | | | | |
| **Subjects in 36-month Group** | | | | | | | | |
| Scan Protocol | #1 | | | | #2 | | | |
| Perinatal Diet | CTR | | WSD | | CTR | | WSD | |
| Post-weaning Diet | CTR | WSD | CTR | WSD | CTR | WSD | CTR | WSD |
| Male | 2 | 3 | 5 | 1 | 1 | 0 | 4 | 0 |
| Female | 2 | 2 | 5 | 4 | 4 | 0 | 2 | 0 |
| Total | 35 | | | | | | | |

Abbreviations: CTR, control diet; WSD, Western-style diet.

Macaque Subject Demographics

MRI scans were acquired in offspring at roughly 4, 6, 11, 21, and 36 months of age. A total of 39, 25, 37, 32, and 35 offspring were included in each age group, with a mean age of 4.41 mo (SD 0.15), 6.59 mo (SD 0.18), 11.04 mo (SD 0.21), 21.10 mo (SD 0.24), and 36.48 mo (SD 0.39) at the time of the scan, respectively. The demographic composition of each age group is summarized in Supplemental Table S1.

Macaque MRI Acquisition, Preprocessing, and Statistical Analysis

The MRI acquisition protocols and preprocessing steps are identical to those described in the main text. The Functional Random Forest (FRF) models for the supplemental classification analyses used the same parameters, input predictors, and outcome measure as described in the main text. A separate FRF model was created for each age group, and the performance metrics were tested separately for each age-specific FRF model. Significance was determined as previously described in the main text.

# RESULTS

## Sensory connections were unable to accurately predict perinatal diet exposure in macaques after 4 months of age

FRF model performance metrics are shown in Supplemental Figure S1. The significant results of the FRF model constructed on the 4 month old offspring data are reported in the main text. The FRF models did not achieve significance across all three performance metrics at any other age point. While there may still be discrete differences in connectivity between perinatal diet groups at these age points, these results indicate that there is not a strong enough pattern of differences in sensory and amygdala connectivity to distinguish between groups.


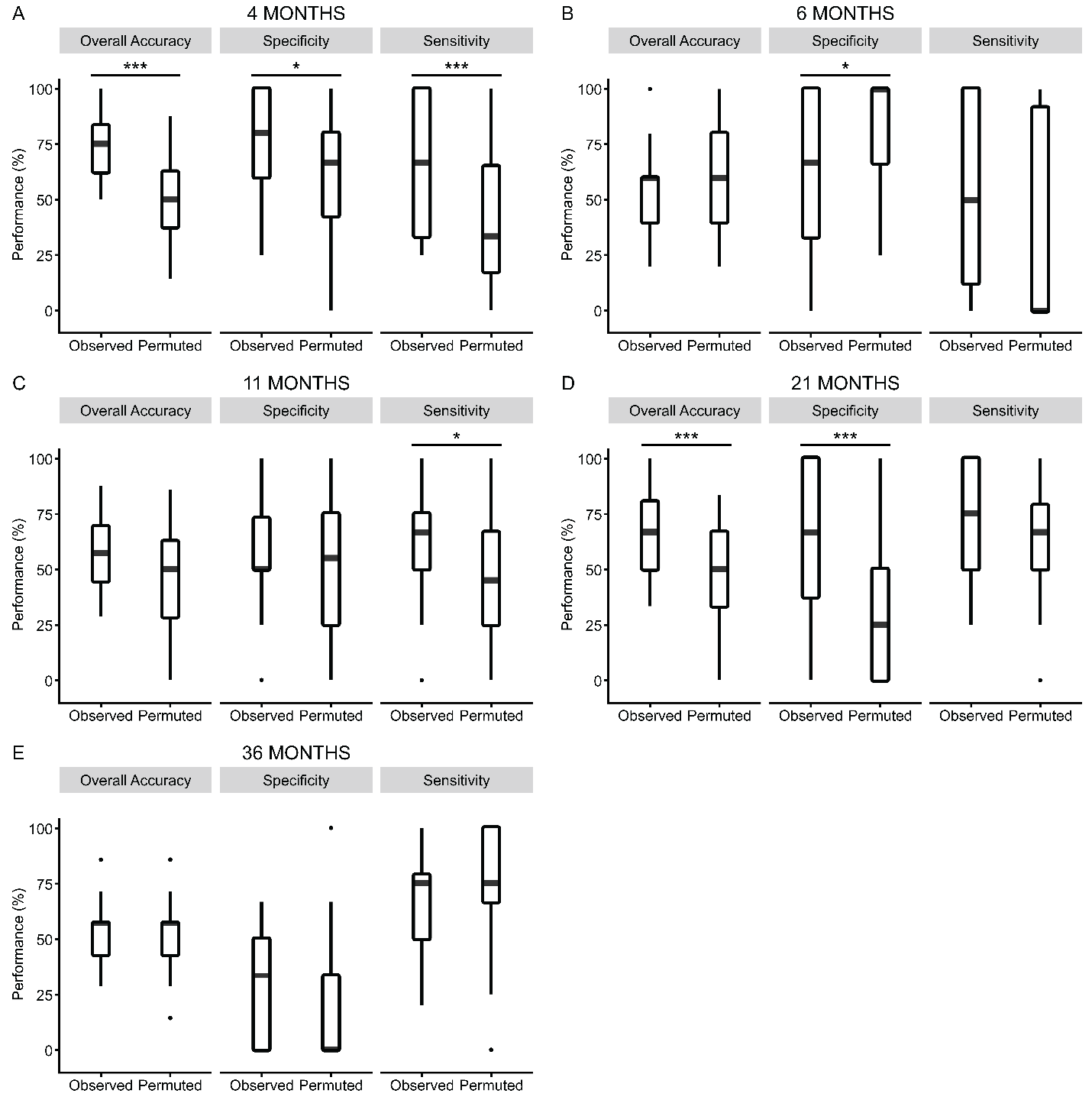


**Figure S1.** Performance metrics for each age-specific functional random forest (FRF) model in macaques. The functional connectivity of 378 connections within and between all sensory network regions and the amygdala were used to predict control (CTR) or Western-style diet (WSD) perinatal diet exposure at 4 (*A*), 6 (*B*), 11 (*C*), 21 (*D*), and 36 (*E*) months of age in macaques. Distributions of overall accuracy, specificity (accurate identification of true negatives when classifying perinatal CTR subjects), and sensitivity (accurate identification of true positives when classifying perinatal WSD subjects) were constructed from the predictions of the observed and permuted models. Statistical significance across all three metrics was required for an age-specific model to be considered valid for predicting perinatal diet group. *A*: a valid model was achieved at 4 months of age (*N* = 39, CTR *n* = 22, female *n* = 19), with the observed model demonstrating a significant improvement in overall accuracy (*P* < 0.001), specificity (*P* = 0.050), and sensitivity (*P* < 0.001). *B*: the model at 6 months of age (*N* = 25, CTR *n* = 16, female *n* = 12) was not valid based on overall accuracy (*P* = 0.601), specificity (*P* = 0.026), and sensitivity (*P* = 0.080). *C*: the model at 11 months of age (*N* = 37, CTR *n* = 19, female *n* = 17) was not valid based on overall accuracy (*P* = 0.062), specificity (*P* = 0.917), and sensitivity (*P* = 0.014). *D*: the model at 21 months of age (*N* = 32, CTR *n* = 13, female *n* = 14) was not valid based on overall accuracy (*P* = 0.001), specificity (*P* < 0.001), and sensitivity (*P* = 0.260). *E*: the model at 36 months of age (*N* = 35, CTR *n* = 14, female *n* = 19) was not valid based on overall accuracy (*P* = 0.519), specificity (*P* = 0.204), and sensitivity (*P* = 0.127). The central line represents the median. Wide bars refer to the 25th/75th percentiles; thinner bars refer to the 2.5th/97.5th percentiles. Significance was determined by a Wilcoxon rank sum test. **P* < 0.05; ****P* < 0.001.

# DISCUSSION

## Functional connectivity may be more susceptible to WSD-associated inflammatory impacts during early infancy due to a potentially stronger maternal immune system response

Perinatal WSD exposure in macaques was associated with differences in functional connectivity at 4 months of age but not at 6, 11, 21, or 36 months of age. Importantly, this may reveal a distinction between prenatal and perinatal WSD exposure. Integral to this distinction is the observation that the juvenile offspring were exposed to their perinatal diet through lactation and solid food consumption until weaning at 7.22 months of age (SD 1.62) for the CTR group and 7.50 months of age (SD 1.14) for the WSD group. Thus, the exposure from the *in utero* environment matched the dietary exposure at both the 4 and 6 month old time points, yet differences in functional connectivity were only strong enough to drive model prediction at 4 months of age. If prolonged postnatal WSD exposure had the ability to impact connectivity strongly, then differences should have been greater after an additional two months of exposure when scans were acquired at a mean of 6.59 months of age (SD 0.18) (3).

This discrepancy highlights the possibility that the maternal environment associated with WSD consumption may lead to *in utero* impacts that differ from the impacts of postnatal WSD exposure. For example, it is well-established that WSD consumption during pregnancy can lead to an elevated inflammatory state (4), whereas the immune system in neonates and infants is still developing (5, 6). Thus, the strong inflammatory response to WSD consumption generated in the dam may differ significantly from the potentially weaker response generated in the offspring during postnatal WSD exposure. Neuroinflammation may impact microglia-mediated processes that shape neural circuit formation, such as neural precursor cell phagocytosis during the third trimester and synaptic pruning during early childhood (7, 8), so a difference in neuroinflammatory influences between the prenatal and postnatal periods may explain the lack of continued impacts at 6 and 11 months of age despite current and recent WSD exposure.

Additionally, prenatal WSD exposure may play a greater role in altering circuitry than early postnatal WSD exposure due to the direct impacts of the inflammatory maternal environment during the most dynamic, and therefore most vulnerable, period of connectome development. The developmental stage of a 4 month old rhesus macaque is roughly equivalent to that of an 8 month old human, whereas 6 and 11 months of age in macaques correspond to 15 and 36 months in humans, respectively (9, 10). The 4 month time point occurs within the period of dynamic circuit formation and may be near enough to the prenatal inflammatory exposure that effects can still be detected by the FRF model, whereas six months would appear to be enough time for the trajectory of functional connectivity to recover. In fact, this trajectory of connectivity is consistent with the trajectory of neuroinflammation found in this non-human primate (NHP) model, indicating that a relationship could exist between the two domains. Prior work has revealed an elevated neuroinflammatory state in fetal WSD offspring, which could have contributed to the differences in connectivity found at 4 months of age in the present study, followed by a slightly suppressed inflammatory state in WSD offspring at 11 months of age, which, if inflammation alters connectivity, is also consistent with the null findings at 11 months in the present study (4, 11, 12).

An important limitation to these proposed mechanisms and conclusions is that the smaller sample size at the 6 month time point could have contributed to the poor model performance. Despite this caveat, the continued inability of the FRF to distinguish between groups at later ages suggests that functional connectivity is plastic and potentially robust against long-lasting impacts from earlier periods of WSD exposure. Future research should seek to corroborate the present findings and, if confirmed, investigate the mechanistic changes that allow the pronounced differences at earlier time points to rapidly resolve.

## Impacts to functional connectivity at later ages may be too weak or heterogeneous to drive model performance

Although the present findings only indicate strong impacts to functional connectivity at the 4 month time point, the null model findings at the later time points do not necessarily rule out the possibility of altered connectivity at these ages. For example, it is possible that only a subset of the WSD offspring developed the characteristics of autism spectrum disorder (ASD) and sensory processing disorder (SPD), and the remaining WSD offspring may have displayed behavior and functional connectivity that was more similar to that of the control subjects. Strong impacts to this subset of WSD offspring may have driven the differences that were detected by the model at 4 months of age, but if those impacts lessened over time, then the connectivity profile across the entire WSD group might no longer differ substantially from that of the control subjects. This would erode the ability of the machine learning model to distinguish between groups, but it does not confirm that the most impacted WSD offspring no longer experienced differences in functional connectivity. Repeating the FRF with the exclusion of subjects in the WSD group that did not display the component behaviors of ASD might improve group-wide consistency and increase model sensitivity, as long as a large enough sample size was retained. The FRF could also be replaced with a more detailed analysis of each connection to test for discrete differences between groups, as this would ensure that individual effects would not be diluted or overpowered by a majority of connections that were not significantly different between groups.

Alternatively, it is possible that the entire group of perinatal WSD offspring continued to experience strong impacts to connectivity across development, but in different ways. There is substantial heterogeneity within ASD, and associations among the diversity of features can often be used to identify subgroups (13-15). Subtypes are also found in typically developing populations, as typical samples experience variance across facets like cognitive ability and genetic background, as well (14, 15). Thus, it is possible that multiple subgroups may have emerged at later ages. This is especially likely given that the consistency of dietary exposures within each group diminished over time. Whereas 100% of the perinatal CTR and WSD groups had only been exposed to their experimental diet by 4 months of age, 21.1% and 77.8% of the 11 month old perinatal CTR and WSD groups, respectively, had switched to the opposite experimental diet after weaning. Although a roughly equal proportion of subjects from each group consumed a post-weaning WSD, thereby ensuring that the model would not be able to distinguish between groups based on differences inherent to the post-weaning WSD, this inconsistency in dietary exposure introduced a within-group difference that may have contributed to the development of subgroups with diverging patterns of connectivity. Thus, while the null results may indicate that the WSD group reverted to the connectivity profile of controls over time, it is also possible that the models were unable to accurately classify these subjects against permuted data because the connectivity patterns within each group were highly variable, inconsistent, and partially overlapping with the connectivity patterns seen in the other group.

Another major limitation to the analysis is the limited sample sizes, which can lead to insufficient model training. Models were trained on a larger, independent sample in humans to attempt to overcome this issue, but these models were also unsuccessful. Although this lends some validation to the findings across species, it also means that the potential limitation of sample size remains. Greatly increasing the sample sizes may reduce group variance and allow the models to learn to detect the various connectivity patterns that characterize the WSD group and distinguish them from the patterns typical of the CTR group.

## Limitations

In addition to the limitations presented above and in the main text, variability among offspring may have been greater at the later ages due to the introduction of other postnatal environmental factors. These factors included the change in post-weaning diet for some subjects, but they also included the change in housing environment after weaning, peer social interactions, stress, and differences in temperament. This study did not control for these factors, so it is possible that the lack of significant results after 4 months of age is attributable to increased environmental variability at later ages rather than a unique susceptibility to perinatal impacts at 4 months.

# REFERENCES

1. **Ramirez JSB, Graham AM, Thompson JR, Zhu JY, Sturgeon D, Bagley JL, Thomas E, Papadakis S, Bah M, Perrone A, Earl E, Miranda-Dominguez O, Feczko E, Fombonne EJ, Amaral DG, Nigg JT, Sullivan EL, Fair DA**. Maternal Interleukin-6 Is Associated With Macaque Offspring Amygdala Development and Behavior. *Cereb Cortex* 30: 1573-1585, 2020. doi:10.1093/cercor/bhz188.

2. **Ramirez JSB, Hermosillo R, Thomas E, Zhu JY, Sturgeon D, Schifsky E, Galassi A, Thompson JR, Bagley JL, Milham MP, Miranda-Dominguez O, Papadakis S, Bah M, Mitchell A, Xu T, Graham AM, Feczko E, Sullivan EL, Fair DA**. Vertex-wise characterization of Non-Human Primate cortical development with prenatal insights. *bioRxiv* 2021. doi:10.1101/2021.09.23.461551.

3. **Miranda-Dominguez O, Ramirez JSB, Mitchell AJ, Perrone A, Earl E, Carpenter S, Feczko E, Graham A, Jeon S, Cohen NJ, Renner L, Neuringer M, Kuchan MJ, Erdman JW, Jr., Fair D**. Carotenoids improve the development of cerebral cortical networks in formula-fed infant macaques. *Sci Rep* 12: 15220, 2022. doi:10.1038/s41598-022-19279-1.

4. **Grayson BE, Levasseur PR, Williams SM, Smith MS, Marks DL, Grove KL**. Changes in melanocortin expression and inflammatory pathways in fetal offspring of nonhuman primates fed a high-fat diet. *Endocrinology* 151: 1622-1632, 2010. doi:10.1210/en.2009-1019.

5. **Basha S, Surendran N, Pichichero M**. Immune responses in neonates. *Expert Rev Clin Immunol* 10: 1171-1184, 2014. doi:10.1586/1744666x.2014.942288.

6. **Christensen LB, Woods TA, Carmody AB, Caughey B, Peterson KE**. Age-related differences in neuroinflammatory responses associated with a distinct profile of regulatory markers on neonatal microglia. *J Neuroinflammation* 11: 70, 2014. doi:10.1186/1742-2094-11-70.

7. **Cunningham CL, Martínez-Cerdeño V, Noctor SC**. Microglia regulate the number of neural precursor cells in the developing cerebral cortex. *J Neurosci* 33: 4216-4233, 2013. doi:10.1523/jneurosci.3441-12.2013.

8. **Eltokhi A, Janmaat IE, Genedi M, Haarman BCM, Sommer IEC**. Dysregulation of synaptic pruning as a possible link between intestinal microbiota dysbiosis and neuropsychiatric disorders. *J Neurosci Res* 98: 1335-1369, 2020. doi:10.1002/jnr.24616.

9. **Workman AD, Charvet CJ, Clancy B, Darlington RB, Finlay BL**. Modeling transformations of neurodevelopmental sequences across mammalian species. *J Neurosci* 33: 7368-7383, 2013. doi:10.1523/jneurosci.5746-12.2013.

10. **Charvet CJ, Clancy B, Finlay BL**. Translating Time across developing mammalian brains: Translate [Online]. Translating Time. https://www.translatingtime.org/translate/ [December 23, 2022].

11. **Papadakis S, Thompson JR, Feczko E, Miranda-Dominguez O, Dunn GA, Selby M, Mitchell AJ, Sullivan EL, Fair DA**. Perinatal Western-style diet exposure associated with decreased microglial counts throughout the arcuate nucleus of the hypothalamus in Japanese macaques. *J Neurophysiol* 131: 241-260, 2024. doi:10.1152/jn.00213.2023.

12. **Dunn GA, Mitchell AJ, Selby M, Fair DA, Gustafsson HC, Sullivan EL**. Maternal diet and obesity shape offspring central and peripheral inflammatory outcomes in juvenile non-human primates. *Brain Behav Immun* 102: 224-236, 2022. doi:10.1016/j.bbi.2022.02.024.

13. **Feczko E, Balba NM, Miranda-Dominguez O, Cordova M, Karalunas SL, Irwin L, Demeter DV, Hill AP, Langhorst BH, Grieser Painter J, Van Santen J, Fombonne EJ, Nigg JT, Fair DA**. Subtyping cognitive profiles in Autism Spectrum Disorder using a Functional Random Forest algorithm. *Neuroimage* 172: 674-688, 2018. doi:10.1016/j.neuroimage.2017.12.044.

14. **Cordova M, Shada K, Demeter DV, Doyle O, Miranda-Dominguez O, Perrone A, Schifsky E, Graham A, Fombonne E, Langhorst B, Nigg J, Fair DA, Feczko E**. Heterogeneity of executive function revealed by a functional random forest approach across ADHD and ASD. *Neuroimage Clin* 26: 102245, 2020. doi:10.1016/j.nicl.2020.102245.

15. **Feczko E, Miranda-Dominguez O, Marr M, Graham AM, Nigg JT, Fair DA**. The Heterogeneity Problem: Approaches to Identify Psychiatric Subtypes. *Trends Cogn Sci* 23: 584-601, 2019. doi:10.1016/j.tics.2019.03.009.

[Page intentionally left blank]

# SUPPLEMENTAL TABLE S2

**Table S2.** Regions of interest included in the functional connectivity analyses.

| **Network** | **ROI Name** | **ROI Abbreviation** |
| --- | --- | --- |
| Auditory | Primary Auditory Cortex | A1 |
| Auditory | Secondary Auditory Cortex | A2 |
| Limbic | Amygdala | Amyg |
| Somatomotor | Posterior Insula | Ip |
| Somatomotor | Primary Somatosensory Cortex | S1 |
| Somatomotor | Secondary Somatosensory Cortex | S2 |
| Somatomotor | Superior Parietal Cortex | PCs |
| Somatomotor | Primary Motor Cortex | M1 |
| Somatomotor | Medial Premotor Cortex | PMCm |
| Somatomotor | Anterior Cingulate Gyrus | CCa |
| Visual | Visual Area 1 | V1 |
| Visual | Visual Area 2 | V2 |
| Visual | Visual Anterior Cortex, Ventral Part | VACv |
| Visual | Visual Anterior Cortex, Dorsal Part | VACd |

The 28 ROIs selected for this study consisted of all ROIs from the auditory, somatomotor, and visual networks, as well as the amygdala ROI from the limbic network, from both brain hemispheres as identified in the Bezgin Regional Map parcellation (1). The 14 ROIs listed in the table summarize these regions from one hemisphere. Abbreviations: ROI, region of interest.

# REFERENCES

1. **Bezgin G, Vakorin VA, van Opstal AJ, McIntosh AR, Bakker R**. Hundreds of brain maps in one atlas: registering coordinate-independent primate neuro-anatomical data to a standard brain. *Neuroimage* 62: 67-76, 2012. doi:10.1016/j.neuroimage.2012.04.013.

[Page intentionally left blank]

# SUPPLEMENTAL TABLE S3 AND FIG. S2

**MATERIALS AND METHODS**

Macaque Adiposity Measurements and Statistical Analysis

The imaging results from the 36-month age group were additionally used to predict the pre-pregnancy adiposity of each subject’s mother. Adiposity could not be predicted at the other ages due to missing adiposity data. Adiposity was measured with dual-energy X-ray absorptiometry scans as previously described (1). The percent body fat of each dam prior to the pregnancy with the subject was used as the predicted value for the regression analysis. This measure was divided into two categories to additionally create a binary classification measure. Dams with a percent body fat less than or equal to 19.6% were classified as “low adiposity,” and those with greater than 19.6% body fat were classified as “high adiposity.” This cut-off was chosen as it roughly corresponds to a body mass index (BMI) of 30 in humans, where values above 30 are considered “obese.” The mean maternal pre-pregnancy percent body fat was 19.25% (SD 8.43) for the perinatal control diet (CTR) group and 21.37% (SD 10.32) for the perinatal Western-style diet (WSD) group in the present study. The number of subjects classified as being exposed to low and high maternal adiposity across the other demographic groups is represented in Supplemental Table S3.

A regression functional random forest (FRF) model was run on the 36-month old subjects using the 378 functional connections to predict percent body fat. A separate classification FRF model was run on the same subjects and connections to predict maternal adiposity group. Both models implemented 5-fold cross-validation with 6 repetitions, 1000 trees, and 90% of the data reserved for the training set, consistent with the parameters of the models used for the longitudinal analysis of perinatal WSD exposure. Significance was determined as previously described in the main text.

**Table S3.** Demographic composition of macaque subjects at the 36 month time point that were exposed to maternal low or high adiposity.

| **Subjects in 36-month Group Classified by Maternal Adiposity Exposure (Low, High Adiposity)** | | | | | | | | |
| --- | --- | --- | --- | --- | --- | --- | --- | --- |
| Scan Protocol | #1 | | | | #2 | | | |
| Perinatal Diet | CTR | | WSD | | CTR | | WSD | |
| Post-weaning Diet | CTR | WSD | CTR | WSD | CTR | WSD | CTR | WSD |
| Male | 2, 0 | 2, 1 | 5, 0 | 1, 0 | 0, 1 | 0, 0 | 0, 4 | 0, 0 |
| Female | 2, 0 | 2, 0 | 1, 4 | 4, 0 | 0, 4 | 0, 0 | 0, 2 | 0, 0 |
| Total | 35 | | | | | | | |

Abbreviations: CTR, control diet; WSD, Western-style diet.

# RESULTS

## Connections between sensory networks and the amygdala do not predict maternal adiposity at 36 months of age

Maternal adiposity, represented as percent body fat in a regression model and as binary categories in a classification model, was not predicted by the 378 connections between sensory networks and the amygdala at 36 months of age. Performance metrics for the FRF regression model, including the Pearson’s correlation coefficient (R), intraclass-correlation coefficient (ICC), and mean absolute error (MAE), were not significantly different between the observed and null (permuted data) models (*P* > 0.05 for each metric, Student’s *t* test). Supplemental Figure S2 demonstrates the weak, negative correlation between observed and predicted maternal body fat percentages for each subject, indicating the inability of the model to accurately predict maternal adiposity. Performance metrics for the FRF classification model were similarly poor, with the average overall accuracy, specificity, and sensitivity metrics lower for the observed model than for the null model (*P* > 0.05 for each metric, Wilcoxon rank sum test). As neither model demonstrated significant predictive capabilities, these models are not valid for predicting a subject’s exposure to maternal adiposity from the functional connectivity within and between their sensory networks and the amygdala.


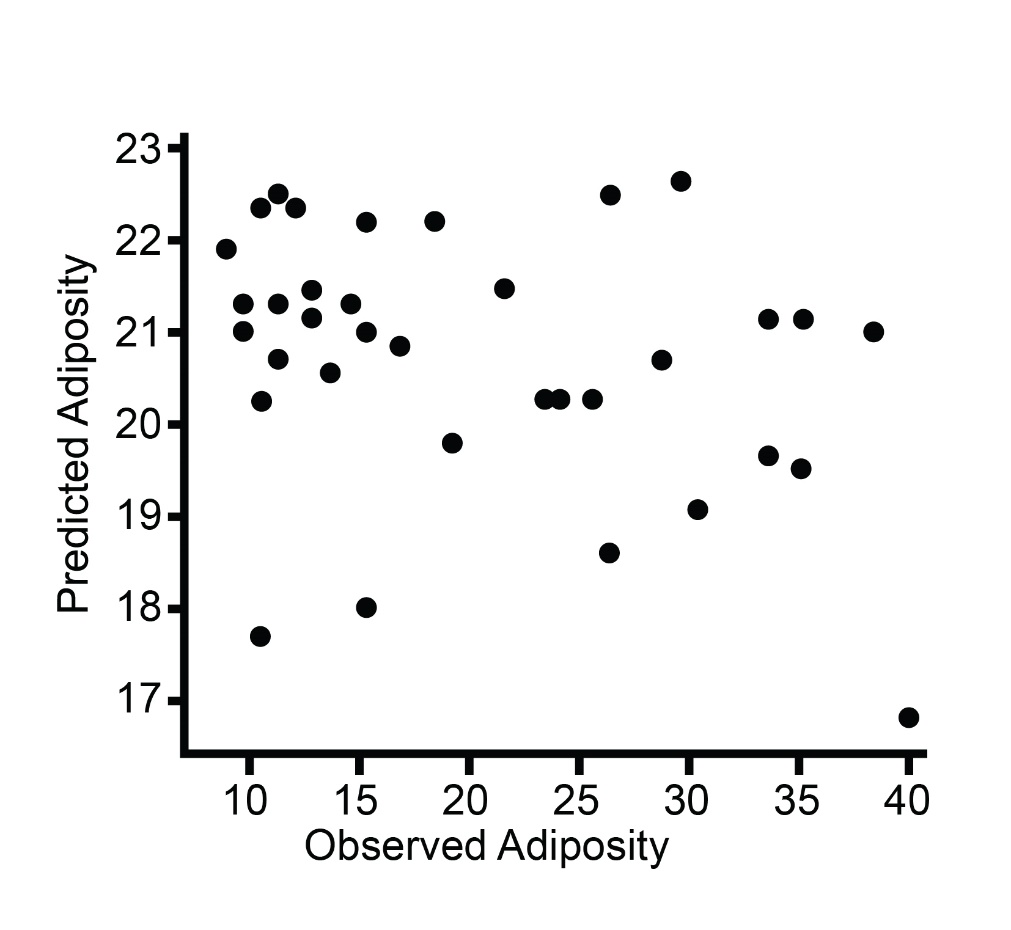


**Figure S2.** Adiposity predictions from the macaque functional random forest (FRF) regression model at 36 months of age. A weak, negative correlation is demonstrated between the observed and predicted maternal pre-pregnancy adiposity values for each subject at 36 months of age (*N* = 35, CTR *n* = 14, female *n* = 19). Adiposity is represented as percent body fat. Performance metrics for the FRF regression model, including the R, ICC, and MAE, were not significantly different between the observed and null (permuted data) models (*P* > 0.05 for each metric). Significance was determined by a Student’s *t* test. Abbreviations: CTR, control diet; ICC, intraclass-correlation coefficient; MAE, mean absolute error; R, Pearson’s correlation coefficient.

# DISCUSSION

## Maternal obesity may be an additive or alternative factor to maternal diet regarding impacts to functional connectivity

This study found an association between perinatal WSD and altered functional connectivity at 4 months of age, but it is possible that alternative, related factors may have had a different impact. For example, a WSD often induces an increase in adiposity, and it is possible that the increased adiposity is the downstream mechanism that impacts offspring functional connectivity. Thus, the higher incidence of increased adiposity in the WSD group would be the factor that drives group-level differences that are generally attributed to diet in this scenario. However, there is metabolic variability within the human and macaque populations, such that some individuals on a WSD remain lean and some individuals on a “control” diet develop obesity. Grouping by perinatal diet exposure therefore mixes subjects from different maternal adiposity exposures and dilutes the impact of adiposity. Grouping explicitly by maternal adiposity could instead lead to greater differences in functional connectivity between groups if adiposity is truly the downstream mechanism that impacts functional connectivity. Alternatively, maternal adiposity might not be the underlying mediator of dietary impacts but rather a separate factor that leads to different outcomes. Grouping explicitly by maternal adiposity could still reveal important information about whether these two factors act on neural circuitry in the same way or through different mechanisms. To explore these possibilities, the FRF analysis in 36 month old macaques was repeated using the same subjects and functional connections but a different predicted outcome measure: maternal adiposity. Roughly half of the subjects from each perinatal diet group were exposed to a high level of maternal adiposity, so the two prenatal factors led to distinct groupings. Maternal adiposity was measured as the percent body fat of the dam prior to pregnancy with the subject. However, both a regression model predicting percent body fat and a classification model comparing binary categories of maternal adiposity were unable to achieve significant performance metrics (Supplemental Fig. S2). This indicates that neither perinatal WSD exposure nor maternal adiposity were associated with altered functional connectivity of sensory and emotional processing areas at 36 months of age. This result adds confidence to the conclusion that the neural circuitry of the selected areas is relatively robust against long-term environmental impacts from the prenatal period.

# REFERENCES

1. **Dunn GA, Mitchell AJ, Selby M, Fair DA, Gustafsson HC, Sullivan EL**. Maternal diet and obesity shape offspring central and peripheral inflammatory outcomes in juvenile non-human primates. *Brain Behav Immun* 102: 224-236, 2022. doi:10.1016/j.bbi.2022.02.024.

[Page intentionally left blank]

# SUPPLEMENTAL TABLE S4 AND FIGS. S3 AND S4

**MATERIALS AND METHODS**

Macaque Amygdala Marker of Neuroinflammation and Statistical Analysis

The imaging results from the 36-month age group were additionally used to predict the number of microglia and macrophages in the amygdala of each subject. This neuroinflammatory measure was collected for only a subset of subjects at 36 months of age following neuroimaging, so the analysis could not be conducted at other time points. As only 10 subjects had functional connectivity and amygdala staining data, results should be considered preliminary.

Following imaging, the number of microglia and macrophages, identified by immunofluorescent staining of ionized calcium-binding adaptor protein-1 (Iba1), was quantified as previously described (Dunn et al., 2022). Images were collected across 7 subregions of the amygdala. The average number of Iba1-stained cells per standard-sized image was calculated across all images collected at each subregion for each subject. Every subject had images from at least five of the seven amygdala subregions. This yielded between five and seven average counts per subject. The average of these average counts was calculated. This final value was considered the average number of Iba1-stained cells across the entire amygdala for that subject.

The demographic composition of the subjects included in this analysis is represented in Supplemental Table S4. Offspring that were exposed to a perinatal Western-style diet (WSD) had a slightly greater number of Iba1-stained cells across the amygdala (Supplemental Fig. S3), with a group average of 23.6 cells (SD 1.2) compared to 21.3 cells (SD 2.7) for controls (CTR). This difference was not statistically significant (*P* = 0.151, Wilcoxon rank sum test).

**Table S4.** Demographic composition of macaque subjects at the 36 month time point that were included in the amygdala staining analysis.

| **Subjects in 36-month Group with the Amygdala Neuroinflammatory Measure** | | | | | | | | |
| --- | --- | --- | --- | --- | --- | --- | --- | --- |
| Scan Protocol | #1 | | | | #2 | | | |
| Perinatal Diet | CTR | | WSD | | CTR | | WSD | |
| Post-weaning Diet | CTR | WSD | CTR | WSD | CTR | WSD | CTR | WSD |
| Male | 1 | 0 | 1 | 0 | 1 | 0 | 1 | 0 |
| Female | 2 | 0 | 1 | 0 | 1 | 0 | 2 | 0 |
| Total | 10 | | | | | | | |

Abbreviations: CTR, control diet; WSD, Western-style diet.


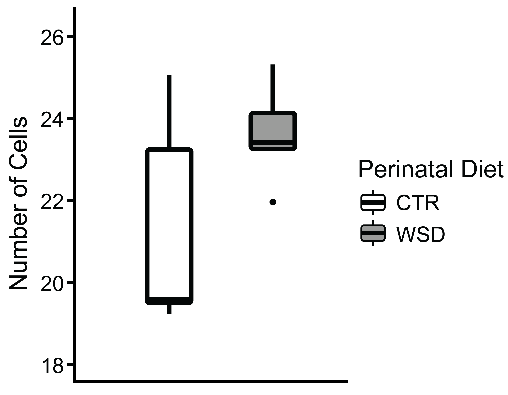


**Figure S3.** Iba1-stained cell count in the amygdala at 36 months. The distribution of the number of Iba1-stained microglia and macrophages per image averaged across the amygdala trended slightly higher for offspring exposed to a perinatal WSD (*n* = 5, female *n* = 3) compared to controls exposed to a perinatal CTR diet (*n* = 5, female *n* = 3) at 36 months of age (*P* = 0.151, Wilcoxon rank sum test). The central line represents the median. Wide bars refer to the 25th/75th percentiles; thinner bars refer to the 2.5th/97.5th percentiles. Abbreviations: CTR, control diet; Iba1, ionized calcium-binding adaptor protein-1; WSD, Western-style diet.

All connections strictly between the amygdala and the sensory networks, totaling 53 connections including the connection between both hemispheres of the amygdala, were selected as training features for a functional random forest (FRF) regression model. The model implemented 6-fold cross-validation with 5 repetitions, 3000 trees, and 90% of the data reserved for the training set. These parameters differed from those used in the longitudinal analysis of perinatal WSD exposure with the goal of mitigating the high variance from the smaller sample size. Significance was determined as previously described in the main text.

Additionally, all connections between and within the sensory areas and the amygdala, totaling 378 connections, were used to predict the average Iba1-stained cell count across the amygdala using a partial least squares regression (PLSR).

# RESULTS

## Amygdala connectivity does not predict amygdala neuroinflammation at 36 months of age

Measures of amygdala connectivity and neuroinflammation were collected in juvenile macaques at 36 months of age (*N* = 10) to explore a potential relationship between neuroinflammation and altered neural circuitry in a region implicated in sensory processing disorder (SPD). The connectivity strengths of the 53 connections between the amygdala and all regions of interest (ROIs) within the sensory networks (Bezgin et al., 2012) were used to train FRF model prediction. The predicted outcome variable was the average number of Iba1-stained microglia and macrophages across the amygdala of each subject. A strong, positive correlation between observed and predicted Iba1-stained cell counts would have demonstrated model accuracy, but this was not achieved by the model (Supplemental Fig. S4). Performance metrics for the FRF regression model, including the Pearson’s correlation coefficient (R), intraclass-correlation coefficient (ICC), and mean absolute error (MAE), were not significantly different between the observed and null (permuted data) models (*P* > 0.05 for each metric, Student’s *t* test). Thus, the model was unable to use amygdala connectivity to predict a measure of neuroinflammation in the amygdala of offspring.


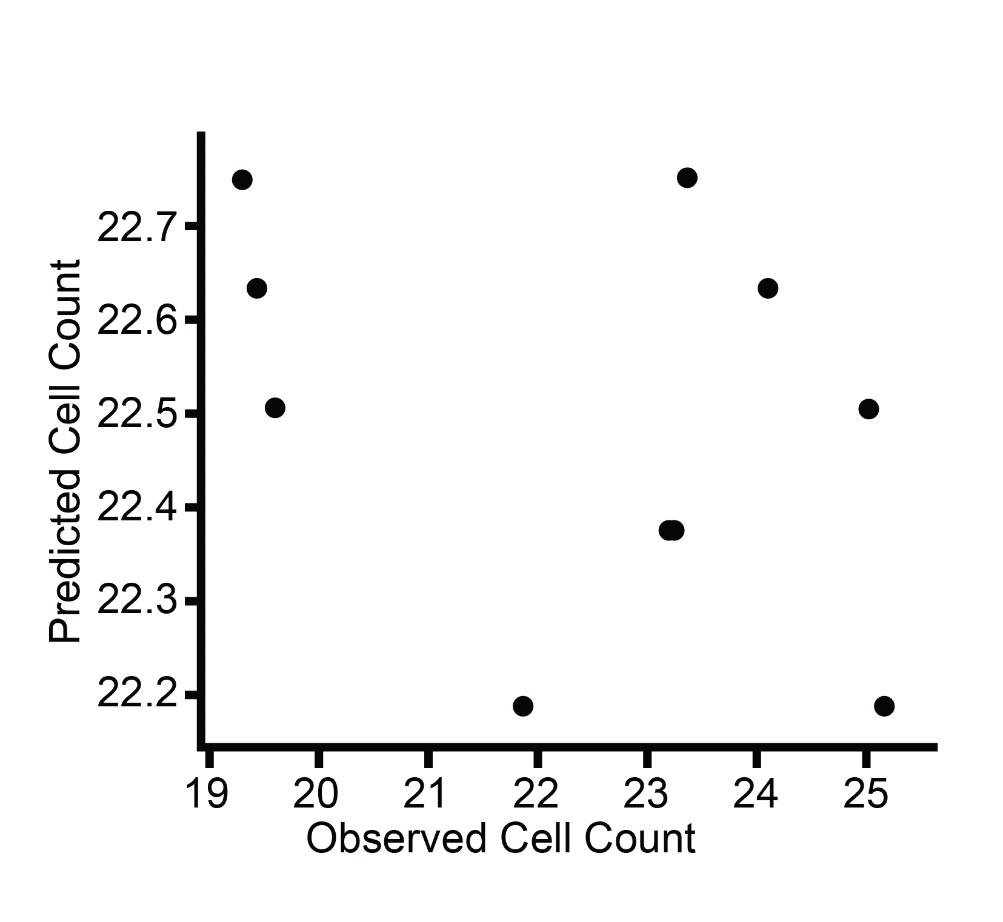


**Figure S4.** Amygdala Iba1-stained cell count predictions from the macaque functional random forest (FRF) regression model at 36 months of age. The predicted number of Iba1-stained microglia and macrophages across the amygdala is compared to the observed number for each subject at 36 months of age (*N* = 10, CTR *n* = 5, female *n* = 6). The weak, negative correlation between observed and predicted Iba1-stained cell count demonstrates the inability of the model to accurately predict a marker of amygdala neuroinflammation from amygdala connectivity strengths. Performance metrics for the FRF regression model, including the R, ICC, and MAE, were not significantly different between the observed and null (permuted data) models (*P* > 0.05 for each metric, Student’s t test). Abbreviations: CTR, control diet; Iba1, ionized calcium-binding adaptor protein-1; ICC, intraclass-correlation coefficient; MAE, mean absolute error; R, Pearson’s correlation coefficient.

Additionally, a PLSR was used to determine whether network groupings within the broader set of 378 connections between and within the sensory areas and the amygdala could predict average amygdala Iba1-stained cell count. As only 10 subjects were available for this analysis, a within-sample approach was used to maximize the available data. This approach revealed that several network groupings were able to predict cell count, as determined by a strong correlation between observed and predicted cell count. The network groupings with an *R* of 1.0 included somatomotor-auditory connections (8 components, *P* < 0.01), somatomotor-somatomotor connections (5 components, *P* < 0.01), amygdala-somatomotor connections (6 components, *P* < 0.01), and somatomotor-visual connections (5 components, *P* < 0.01), indicating that these connections were the most predictive of cell count. The connectivity within the visual-visual and visual-auditory network groupings similarly generated models that strongly predicted cell count with *R* values of 0.88 and 0.86 respectively (2 components and *P* < 0.01 for each). The auditory-auditory, amygdala-visual, and amygdala-auditory network groupings, however, were less predictive of cell count (*R* = 0.62, 0.61, and 0.59, respectively, for 2, 1, and 1 component, respectively, with *P* < 0.05 for all), indicating that there was more noise leading to weaker associations within the samples.

The PLSR was repeated using an out-of-sample approach to determine whether these findings were generalizable. Of the 10 subjects, 9 were used to train a model, and the amygdala cell count of the 10^th^ subject was predicted using its functional connectivity. This prediction was repeated once for each subject, yielding 10 model runs per network grouping. The distribution of the mean absolute error of these predictions was compared to that of 1,000 null models wherein the data were randomly permuted. Using this approach, no network grouping achieved a significant or strong *R* value above 0.07 for any number of components. Additionally, the effect size (Cohen’s *d*) between the two distributions of mean absolute error was small for each network grouping, indicating that the ability of the network connections to predict cell count in a novel sample is not meaningfully better than chance. This approach was similarly repeated with the cell count data normalized by a Box-Cox transformation rather than by z-scores, and was repeated while leaving 3 subjects out instead of 1, with similar non-significant findings. Thus, although the connectivity of certain network groupings was able to predict cell count in a data set that had been used to train the model, no network grouping was able to adequately predict cell count in a novel sample, demonstrating that the observed relationship between connectivity and cell count was not generalizable. The number of microglia in the amygdala likely does not have any strong bearing on the connectivity of sensory areas and the amygdala, though a larger sample size would be useful in confirming this finding.

# DISCUSSION

## Maternal inflammation may be an additive or alternative factor to maternal diet regarding impacts to functional connectivity

A potential mediator of altered circuitry related to WSD exposure is neuroinflammation. As mentioned previously, prenatal exposure to maternal inflammation may impact circuit formation *in utero*, but it is also possible for prenatal inflammation to program for long-term postnatal inflammation (Bilbo and Schwarz, 2009; Romero et al., 2007; Denizli et al., 2022). Postnatal inflammation, either deriving from prenatal WSD exposure or other life experiences, could continue to impact circuit formation through the process of synaptic pruning. Synaptic pruning is the process of removing weak connections between neurons. It is conducted by microglia, which are the resident immune cells of the brain. In a state of heightened neuroinflammation, microglia become activated. Research demonstrates that microglial activation can lead to dramatic differences in synaptic pruning activity, which impacts neural circuit formation and behavioral outcomes (Kim et al., 2017; Cowan and Petri, 2018; Kleinhans et al., 2016; Huang et al., 2016; Ypma et al., 2016). Thus, if differences in functional connectivity are able to predict a marker of increased neuroinflammation, then this would suggest that disruption to a microglial-mediated process like synaptic pruning may be responsible for the alterations in circuitry. To explore this possibility, the FRF analysis in 36 month old macaques was repeated using a subset of subjects that had undergone a procedure for measuring neuroinflammation in the amygdala. Increased neuroinflammation can lead to increased microglial proliferation and migration (Sarlus and Heneka, 2017; Belhocine et al., 2022; Lively and Schlichter, 2013), so the number of microglia in the amygdala was quantified for ten subjects from the 36 month imaging group. A subset of 53 connections between the amygdala and all ROIs within the sensory networks was used to predict microglial count with an FRF regression model. The model was unable to achieve significant performance metrics, however, so an association between amygdala connectivity and neuroinflammation could not be drawn (Supplemental Fig. S4). This finding is limited by the small sample size, so further research should seek to expand the analysis to a larger sample. Additionally, it would be interesting to repeat this analysis at the 4 month age point to see whether increased neuroinflammation leads to the same changes in functional connectivity as seen with perinatal WSD exposure. If both factors are associated with similar changes, and if there is high overlap between the perinatal WSD group and the subjects with increased neuroinflammation, this would suggest that perinatal WSD exposure may impact neural circuitry through increased neuroinflammation. Further research could identify whether microglia in particular mediate that relationship.

# REFERENCES

**Belhocine S, Machado Xavier A, Distéfano-Gagné F, Fiola S, Rivest S, Gosselin D**. Context-dependent transcriptional regulation of microglial proliferation. *Glia* 70: 572-589, 2022. doi:10.1002/glia.24124.

**Bezgin G, Vakorin VA, van Opstal AJ, McIntosh AR, Bakker R**. Hundreds of brain maps in one atlas: registering coordinate-independent primate neuro-anatomical data to a standard brain. *Neuroimage* 62: 67-76, 2012. doi:10.1016/j.neuroimage.2012.04.013.

**Bilbo SD, Schwarz JM**. Early-life programming of later-life brain and behavior: a critical role for the immune system. *Front Behav Neurosci* 3: 14, 2009. doi:10.3389/neuro.08.014.2009.

**Cowan M, Petri WA, Jr.** Microglia: Immune Regulators of Neurodevelopment. *Front Immunol* 9: 2576, 2018. doi:10.3389/fimmu.2018.02576.

**Denizli M, Capitano ML, Kua KL**. Maternal obesity and the impact of associated early-life inflammation on long-term health of offspring. *Front Cell Infect Microbiol* 12: 940937, 2022. doi:10.3389/fcimb.2022.940937.

**Dunn GA, Mitchell AJ, Selby M, Fair DA, Gustafsson HC, Sullivan EL**. Maternal diet and obesity shape offspring central and peripheral inflammatory outcomes in juvenile non-human primates. *Brain Behav Immun* 102: 224-236, 2022. doi:10.1016/j.bbi.2022.02.024.

**Huang WC, Chen Y, Page DT**. Hyperconnectivity of prefrontal cortex to amygdala projections in a mouse model of macrocephaly/autism syndrome. *Nat Commun* 7: 13421, 2016. doi:10.1038/ncomms13421.

**Kim HJ, Cho MH, Shim WH, Kim JK, Jeon EY, Kim DH, Yoon SY**. Deficient autophagy in microglia impairs synaptic pruning and causes social behavioral defects. *Mol Psychiatry* 22: 1576-1584, 2017. doi:10.1038/mp.2016.103.

**Kleinhans NM, Reiter MA, Neuhaus E, Pauley G, Martin N, Dager S, Estes A**. Subregional differences in intrinsic amygdala hyperconnectivity and hypoconnectivity in autism spectrum disorder. *Autism Res* 9: 760-772, 2016. doi:10.1002/aur.1589.

**Lively S, Schlichter LC**. The microglial activation state regulates migration and roles of matrix-dissolving enzymes for invasion. *J Neuroinflammation* 10: 75, 2013. doi:10.1186/1742-2094-10-75.

**Romero E, Ali C, Molina-Holgado E, Castellano B, Guaza C, Borrell J**. Neurobehavioral and immunological consequences of prenatal immune activation in rats. Influence of antipsychotics. *Neuropsychopharmacology* 32: 1791-1804, 2007. doi:10.1038/sj.npp.1301292.

**Sarlus H, Heneka MT**. Microglia in Alzheimer's disease. *J Clin Invest* 127: 3240-3249, 2017. doi:10.1172/jci90606.

**Ypma RJ, Moseley RL, Holt RJ, Rughooputh N, Floris DL, Chura LR, Spencer MD, Baron-Cohen S, Suckling J, Bullmore ET, Rubinov M**. Default Mode Hypoconnectivity Underlies a Sex-Related Autism Spectrum. *Biol Psychiatry Cogn Neurosci Neuroimaging* 1: 364-371, 2016. doi:10.1016/j.bpsc.2016.04.006.

[Page intentionally left blank]

# SUPPLEMENTAL TABLE S5 AND FIG. S5

**MATERIALS AND METHODS**

Human Participants and MRI Processing

Data were obtained from the ABCD Study at the baseline time point (year 1 arm 1) when participants were between the ages of 9 and 10 years old (108-131 months). The ABCD Study has been described previously (1-10). Written informed consent and assent were obtained from legal guardians and children, respectively, to participate in the ABCD Study, and the Institutional Review Boards at Oregon Health & Science University and the University of Minnesota approved the analysis of pre-existing neuroimaging data. Participants were included in the present study if they met the following criteria: 1) MRI scans passed quality control; 2) functional data passed motion quality thresholds; 3) the number of frames in the motion and CIFTI files matched; 4) the participant had a sensory over-responsivity (SOR) score. Details on these four criteria follow.

1) MRI scans were collected at 21 sites across the United States and uploaded to the National Institute of Mental Health (NIMH) Data Archive (NDA) (11). Imaging data from this shared repository was included in the present study only if it had passed the quality control standards set by the ABCD Data Analytics and Informatics Core (DAIC), also known as the Data Analysis, Informatics & Resource Center (DAIRC) (4, 12-14). Imaging data were processed using a modified version of the Human Connectome Project (HCP) Pipeline by the Developmental Cognition and Neuroimaging (DCAN) Labs (15-18), now known as the Center for Developmental NeuroImaging (CDNI). The Bezgin Regional Map parcellation (19), which identifies functional network parcellations in macaques, was deformed and registered into human space using a previously described method of joint-embedding to align cortical surfaces across species (20-21). This human-aligned Bezgin parcellation was applied to the functional outputs to label the human brain in the same way as the macaque brain and allow for cross-species ROI-based comparisons.

2) Motion censoring was performed in the same way as described for the macaque imaging data. Frames with FD > 0.2 mm were excluded, as well as the first five frames and any isolated frames that came from a group of fewer than five contiguous frames below this threshold. Exactly 8 minutes of data that passed this motion threshold was extracted. Participants with fewer than 8 minutes of motion-censored data were excluded.

3) The Brain Imaging Connectivity Extraction Program Solution (BICEPS), a MATLAB-based tool (22) developed by the DCAN Labs, was used to perform motion censoring (23-25). After motion censoring, the software extracted the correct number of frames and created a connectivity matrix, or a file that recorded the correlation between the time series signals for every pair of ROIs identified by the Bezgin parcellation. During this process, the number of frames found in the motion numbers file was compared to the number of frames found in the CIFTI parcellated time series file. A mismatch in the number of frames could lead to the inclusion of frames that did not pass the motion quality threshold. Three subjects were excluded due to a mismatched number of frames.

4) The 11-item Short-Social Responsiveness Scale was administered when participants were 9 to 12 years of age. The included participants were interviewed at a mean age of 131.74 mo (SD 7.75). One of the items stated that the participant “Seems overly sensitive to sounds, textures, or smells.” Parents responded to this item with “Not True”, “Sometimes True,” “Often True,” or “Almost Always True.” These responses were converted to an ordinal variable with the levels 1, 2, 3, and 4 respectively. This item was considered to be a measure of SOR and was used as the outcome variable for the analysis. Subjects that did not have an SOR score were excluded.

Of the final set of 6,806 subjects (female *n* = 3,450), 5,670 (female *n* = 2,941) had an SOR score of 1; 805 (female *n* = 376) had a score of 2; 229 (female *n* = 90) had a score of 3; and 102 (female *n* = 43) had a score of 4.

Statistical Analysis of Human Data

The analysis method used for the macaque data was repeated with the human data to better allow for direct cross-species comparisons. Three separate functional random forest (FRF) models were constructed to assess the data in slightly different ways. The first was a regression model, where the four possible SOR scores were treated as a continuous variable for the full set of 6,806 subjects. The second was a classification model where the subjects were grouped into two categories: those with an SOR score of 1 (*n* = 5,670), and those with an SOR score of 2, 3, or 4 (*n* = 1,136). Unlike for regression, it is useful to have balanced group sizes for classification, so the algorithm randomly selected 1,136 subjects from each of the two categories when constructing the model. The third model was also a classification model, but it excluded subjects that had an SOR score of 2. As a result, subjects with a score of 1 (*n* = 5,670) were compared to subjects with a score of 3 or 4 (*n* = 331). As previously described, sample sizes in the thousands are necessary to generate reproducible results from studies that associate brain connectivity with psychiatric symptomology (1). Thus, while a power analysis was not conducted for this study, the regression and first classification models were expected to have an adequate sample size to reveal effects. While the second classification model trained with the smallest number of subjects (random selection of 331 per group), it is possible that connectivity differences between subjects on distal ends of the SOR scale would be pronounced enough to overcome this limitation.

The input predictors for all models consisted of the same 378 features used in the macaque models, and the outcome measure was the SOR score of the human subject. A random selection of 80% of the data was used for training, and the remaining 20% was reserved for testing. Models were tested using three repetitions of 10-fold cross-validation. The regression model was constructed with 1,000 trees, whereas the two classification models were constructed with 2,000 trees each. Model performance for the regression model was determined by improvements to three measures: the mean absolute error (MAE) in predicting the SOR score, the correlation between subjects’ observed and predicted SOR scores (R), and the intraclass-correlation coefficient (ICC). A Student’s t test was used to evaluate the significance of these three performance metrics. Model performance for the classification models was determined by assessing the distributions of overall accuracy, specificity, and sensitivity between the observed and null models with a Wilcoxon rank sum test, as described previously for the macaque analysis.

# RESULTS

## SOR score in children was not predicted by differences in sensory and amygdala connectivity

In addition to exploring the functional connectivity patterns present in macaques exposed to a proposed prenatal predictor of sensory processing disorder (SPD), this study also explored the functional connectivity patterns of humans that exhibited a measure of SPD. This additional analysis is advantageous because results from this independent data set can provide insight into the generalizability of the macaque findings, and the use of a human model of SPD offers maximal translational ability to the animal model. Three FRF models were run on slightly different subsets of human imaging data from the ABCD Study. None of the models demonstrated significant predictive capabilities (Supplemental Table S5, Fig. S5). Thus, these models were not valid for predicting a child’s SOR score from the functional connectivity within and between their sensory networks and the amygdala. This inconsistency in outcomes, where the 4 month old macaque data was able to train a valid model while the human data was not, could be due to differences related to unique brain physiology between species. However, macaque data at the comparable developmental age also yielded an invalid model, suggesting that these null results in the context of SPD may translate across species.

**Table S5.** Performance metrics for the human sensory over-responsivity (SOR) regression model

| Model | R | | ICC | | MAE | |
| --- | --- | --- | --- | --- | --- | --- |
|  | Observed | Permuted | Observed | Permuted | Observed | Permuted |
| Regression | 0.0187 | 0.0010 | 0.5171 | 0.5140 | 0.3946 | 0.3968 |

Performance metrics for the functional random forest (FRF) regression model were not significantly different between the observed and null (permuted data) models (*P* = 0.236, 0.676, and 0.314 for the R, ICC, and MAE metrics, respectively). Values displayed are the means across three model repetitions. Significance was determined by a Student’s *t* test. Abbreviations: ICC, intraclass-correlation coefficient; MAE, mean absolute error; R, Pearson’s correlation coefficient; SOR, sensory over-responsivity.


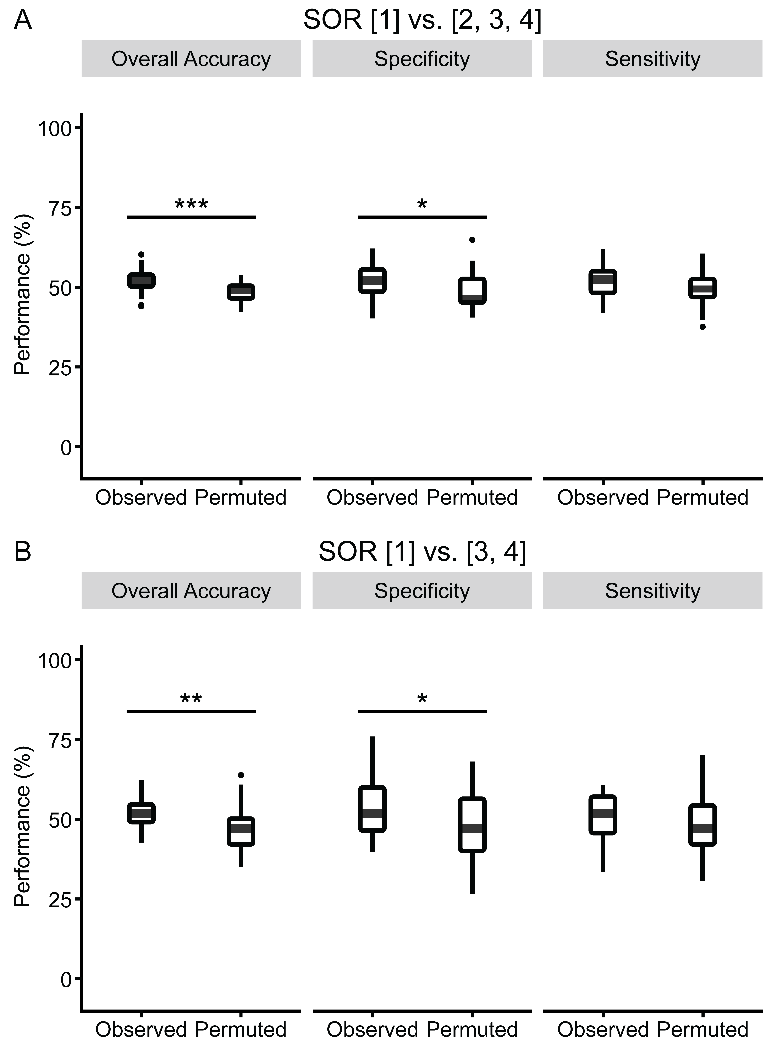


**Figure S5.** Performance metrics for the human sensory over-responsivity (SOR) functional random forest (FRF) classification models. The functional connectivity of 378 connections within and between all sensory network regions and the amygdala were used to predict SOR score in children. Distributions of overall accuracy, specificity (accurate identification of true negatives when classifying subjects with an SOR score of 1), and sensitivity (accurate identification of true positives when classifying subjects with an SOR score of 2, 3, or 4 in *A* and an SOR score of 3 or 4 in *B*) were constructed from the predictions of the observed and permuted models. Statistical significance across all three metrics was required for a model to be considered valid for predicting SOR score. *A*: this classification model predicted two classes of SOR score: one class was defined by a score of “1,” and the other was defined by a score of “2,” “3,” or “4.” The model included data from 6,806 subjects (SOR score of “1” *n* = 5,670, female *n* = 3,450), though the algorithm selected a random sample of 1,136 subjects from the group with an SOR score of “1” to create balanced group sizes during model construction. The model was not valid based on overall accuracy (*P* = 0.001), specificity (*P* = 0.015), and sensitivity (*P* = 0.070). *B*: this classification model predicted two classes of SOR score: one class was defined by a score of “1,” and the other was defined by a score of “3” or “4.” The model included data from 6,001 subjects (SOR score of “1” *n* = 5,670, female *n* = 3,074), though the algorithm selected a random sample of 331 subjects from the group with an SOR score of “1” to create balanced group sizes during model construction. The model was not valid based on overall accuracy (*P* = 0.007), specificity (*P* = 0.034), and sensitivity (*P* = 0.236). The central line represents the median. Wide bars refer to the 25th/75th percentiles; thinner bars refer to the 2.5th/97.5th percentiles. Significance was determined by a Wilcoxon rank sum test. **P* < 0.05; ***P* < 0.01; ****P* < 0.001.

# DISCUSSION

## A cross-species brain parcellation means functional connectivity findings could have been directly translated between the human and macaque cohorts if the FRF models were valid

The FRF analysis performed in macaques was repeated in humans using highly conserved methodology to reduce potential confounds and variance. This included the use of the same minimal MRI preprocessing standards via similar pipelines, the same brain parcellation to label ROIs, the same set of 378 connections within and between sensory areas and the amygdala, and the same FRF algorithm for analysis. Major differences included a much larger sample size in humans (up to 6,806 subjects), higher motion censoring standards in humans (8 min at FD < 0.2 mm instead of 20 minutes at FD < 0.3 mm), and different outcome measures.

The predicted outcome measure in humans was an SOR score that ranged from no observed issues with appearing overly sensitive to sensory stimuli (a score of “1”) to sometimes (“2”), often (“3”), and almost always seeming overly sensitive (“4”). Given that a prenatal Western-style diet (WSD) is a known predictor of the behavioral phenotypes of autism spectrum disorder (ASD)—a neurodevelopmental disorder which is highly comorbid with SPD—and given that the WSD offspring displayed the component behaviors of ASD, the children with an SOR score of 1 were hypothesized to have functional connectivity similar to macaques with perinatal control (CTR) diet exposure, and children with a score of 2, 3, or 4 were hypothesized to show connectivity patterns consistent with the perinatal WSD macaque cohort. If the FRF model that trained on the human data was valid for predicting SOR score from the set of functional connections, then the model could have been applied to the macaque data. The cross-species brain parcellation would enable the identical set of functional connections in macaques to be processed through the human-based decision trees. Each macaque would have been assigned a continuous SOR score or binary SOR class, and a follow-up analysis could have determined whether the perinatal WSD group was assigned a higher SOR score. This would have indicated that the functional connectivity patterns seen in WSD macaques were similar to those of children with SOR, thereby implicating perinatal WSD exposure in the development of SOR. This analytical exploration could not be conducted, however, because none of the variations of the human FRF model achieved significance across all three performance metrics.

Instead, poor model performance indicates that the functional connectivity of the sensory systems and the amygdala did not differ substantially between children with different SOR scores. This result is consistent with the null findings in macaques at the equivalent developmental time point of 36 months of age. While a concrete relationship cannot be established between null results from the macaque and human analyses, this demonstrates a consistent absence of strong, distributed SPD-related impacts to functional connectivity in late childhood. Alternatively, it is also possible that fewer or weaker connectivity differences existed in the human cohort but were not pronounced enough to train the FRF model, or that the single-item SOR score was an unreliable measure of the intended SPD symptom.

# REFERENCES

1. **Marek S, Tervo-Clemmens B, Calabro FJ, Montez DF, Kay BP, Hatoum AS, Donohue MR, Foran W, Miller RL, Hendrickson TJ, Malone SM, Kandala S, Feczko E, Miranda-Dominguez O, Graham AM, Earl EA, Perrone AJ, Cordova M, Doyle O, Moore LA, Conan GM, Uriarte J, Snider K, Lynch BJ, Wilgenbusch JC, Pengo T, Tam A, Chen J, Newbold DJ, Zheng A, Seider NA, Van AN, Metoki A, Chauvin RJ, Laumann TO, Greene DJ, Petersen SE, Garavan H, Thompson WK, Nichols TE, Yeo BTT, Barch DM, Luna B, Fair DA, Dosenbach NUF**. Reproducible brain-wide association studies require thousands of individuals. *Nature* 603: 654-660, 2022. doi:10.1038/s41586-022-04492-9.

2. **Garavan H, Bartsch H, Conway K, Decastro A, Goldstein RZ, Heeringa S, Jernigan T, Potter A, Thompson W, Zahs D**. Recruiting the ABCD sample: Design considerations and procedures. *Dev Cogn Neurosci* 32: 16-22, 2018. doi:10.1016/j.dcn.2018.04.004.

3. **Heeringa SG, Berglund PA**. A Guide for Population-based Analysis of the Adolescent Brain Cognitive Development (ABCD) Study Baseline Data. *bioRxiv* 2020. doi:10.1101/2020.02.10.942011.

4. **Collection 3165 - ABCD-BIDS Community Collection (ABCC)**. ABCD-BIDS Community Collection (ABCC) Documentation Summary [Online]. Read the Docs. <https://collection3165.readthedocs.io/en/stable/> [March 5, 2025].

5. **Hermosillo RJM, Moore LA, Feczko E, Miranda-Domínguez Ó, Pines A, Dworetsky A, Conan G, Mooney MA, Randolph A, Graham A, Adeyemo B, Earl E, Perrone A, Carrasco CM, Uriarte-Lopez J, Snider K, Doyle O, Cordova M, Koirala S, Grimsrud GJ, Byington N, Nelson SM, Gratton C, Petersen S, Feldstein Ewing SW, Nagel BJ, Dosenbach NUF, Satterthwaite TD, Fair DA**. A precision functional atlas of personalized network topography and probabilities. *Nat Neurosci* 27: 1000-1013, 2024. doi:10.1038/s41593-024-01596-5.

6. **Byington N, Grimsrud G, Mooney MA, Cordova M, Doyle O, Hermosillo RJM, Earl E, Houghton A, Conan G, Hendrickson TJ, Ragothaman A, Carrasco CM, Rueter A, Perrone A, Moore LA, Graham A, Nigg JT, Thompson WK, Nelson SM, Feczko E, Fair DA, Miranda-Dominguez O**. Polyneuro risk scores capture widely distributed connectivity patterns of cognition. *Dev Cogn Neurosci* 60: 101231, 2023. doi:10.1016/j.dcn.2023.101231.

7. **Feczko E, Conan G, Marek S, Tervo-Clemmens B, Cordova M, Doyle O, Earl E, Perrone A, Sturgeon D, Klein R, Harman G, Kilamovich D, Hermosillo R, Miranda-Dominguez O, Adebimpe A, Bertolero M, Cieslak M, Covitz S, Hendrickson T, Juliano AC, Snider K, Moore LA, Uriartel J, Graham AM, Calabro F, Rosenberg MD, Rapuano KM, Casey B, Watts R, Hagler D, Thompson WK, Nichols TE, Hoffman E, Luna B, Garavan H, Satterthwaite TD, Ewing SF, Nagel B, Dosenbach NUF, Fair DA**. Adolescent Brain Cognitive Development (ABCD) Community MRI Collection and Utilities. *bioRxiv* 2021. doi:10.1101/2021.07.09.451638.

8. **Day TKM, Hermosillo R, Conan G, Randolph A, Perrone A, Earl E, Byington N, Hendrickson TJ, Elison JT, Fair DA, Feczko E**. Multi-level fMRI analysis applied to hemispheric specialization in the language network, functional areas, and their behavioral correlations in the ABCD sample. *Dev Cogn Neurosci* 66: 101355, 2024. doi:10.1016/j.dcn.2024.101355.

9. **Mooney MA, Hermosillo RJM, Feczko E, Miranda-Dominguez O, Moore LA, Perrone A, Byington N, Grimsrud G, Rueter A, Nousen E, Antovich D, Feldstein Ewing SW, Nagel BJ, Nigg JT, Fair DA**. Cumulative Effects of Resting-State Connectivity Across All Brain Networks Significantly Correlate with Attention-Deficit Hyperactivity Disorder Symptoms. *J Neurosci* 44: 2024. doi:10.1523/jneurosci.1202-23.2023.

10. **Holt-Gosselin B, Keding TJ, Rodrigues K, Rueter A, Hendrickson TJ, Perrone A, Byington N, Houghton A, Miranda-Dominguez O, Feczko E, Fair DA, Joormann J, Gee DG**. Familial risk for depression moderates neural circuitry in healthy preadolescents to predict adolescent depression symptoms in the Adolescent Brain Cognitive Development (ABCD) Study. *Dev Cogn Neurosci* 68: 101400, 2024. doi:10.1016/j.dcn.2024.101400.

11. **NIMH Data Archive**. ABCD Data Repository [Online]. National Institutes of Health. <https://nda.nih.gov/abcd>. [March 5, 2025].

12. **Saragosa-Harris NM, Chaku N, MacSweeney N, Guazzelli Williamson V, Scheuplein M, Feola B, Cardenas-Iniguez C, Demir-Lira E, McNeilly EA, Huffman LG, Whitmore L, Michalska KJ, Damme KS, Rakesh D, Mills KL**. A practical guide for researchers and reviewers using the ABCD Study and other large longitudinal datasets. *Dev Cogn Neurosci* 55: 101115, 2022. doi:10.1016/j.dcn.2022.101115.

13. **Hagler DJ, Jr., Hatton S, Cornejo MD, Makowski C, Fair DA, Dick AS, Sutherland MT, Casey BJ, Barch DM, Harms MP, Watts R, Bjork JM, Garavan HP, Hilmer L, Pung CJ, Sicat CS, Kuperman J, Bartsch H, Xue F, Heitzeg MM, Laird AR, Trinh TT, Gonzalez R, Tapert SF, Riedel MC, Squeglia LM, Hyde LW, Rosenberg MD, Earl EA, Howlett KD, Baker FC, Soules M, Diaz J, de Leon OR, Thompson WK, Neale MC, Herting M, Sowell ER, Alvarez RP, Hawes SW, Sanchez M, Bodurka J, Breslin FJ, Morris AS, Paulus MP, Simmons WK, Polimeni JR, van der Kouwe A, Nencka AS, Gray KM, Pierpaoli C, Matochik JA, Noronha A, Aklin WM, Conway K, Glantz M, Hoffman E, Little R, Lopez M, Pariyadath V, Weiss SR, Wolff-Hughes DL, DelCarmen-Wiggins R, Feldstein Ewing SW, Miranda-Dominguez O, Nagel BJ, Perrone AJ, Sturgeon DT, Goldstone A, Pfefferbaum A, Pohl KM, Prouty D, Uban K, Bookheimer SY, Dapretto M, Galvan A, Bagot K, Giedd J, Infante MA, Jacobus J, Patrick K, Shilling PD, Desikan R, Li Y, Sugrue L, Banich MT, Friedman N, Hewitt JK, Hopfer C, Sakai J, Tanabe J, Cottler LB, Nixon SJ, Chang L, Cloak C, Ernst T, Reeves G, Kennedy DN, Heeringa S, Peltier S, Schulenberg J, Sripada C, Zucker RA, Iacono WG, Luciana M, Calabro FJ, Clark DB, Lewis DA, Luna B, Schirda C, Brima T, Foxe JJ, Freedman EG, Mruzek DW, Mason MJ, Huber R, McGlade E, Prescot A, Renshaw PF, Yurgelun-Todd DA, Allgaier NA, Dumas JA, Ivanova M, Potter A, Florsheim P, Larson C, Lisdahl K, Charness ME, Fuemmeler B, Hettema JM, Maes HH, Steinberg J, Anokhin AP, Glaser P, Heath AC, Madden PA, Baskin-Sommers A, Constable RT, Grant SJ, Dowling GJ, Brown SA, Jernigan TL, Dale AM**. Image processing and analysis methods for the Adolescent Brain Cognitive Development Study. *Neuroimage* 202: 116091, 2019. doi:10.1016/j.neuroimage.2019.116091.

14. **ABCD Study**. Data Analysis, Informatics & Resource Center (DAIRC) [Online]. ABCD Study. <https://abcdstudy.org/study-sites/daic/>. [March 3, 2025].

15. **Sturgeon D, Earl E, Madison T, Perrone A, Kathy, Rueter A, hough129, Rockets2theMoon**. DCAN-Labs/abcd-hcp-pipeline [Online]. Zenodo. doi.org/10.5281/zenodo.2587209. [December 23, 2022].

16. **Center for Developmental NeuroImaging (CDNI)**. DCAN-Labs/abcd-hcp-pipeline: release v0.0.0 [Online]. GitHub. <https://github.com/DCAN-Labs/abcd-hcp-pipeline/releases/tag/v0.0.0>. [March 5, 2025].

17. **ABCD Data Analytics and Informatics Resource Center**. ABCD-STUDY/abcd-hcp-pipeline: v0.0.0 [Online]. GitHub. <https://github.com/ABCD-STUDY/abcd-hcp-pipeline/releases/tag/v0.0.0>. [March 5, 2025].

18. **Collection 3165 - ABCD-BIDS Community Collection (ABCC)**. Pipeline [Online]. Read the Docs. <https://collection3165.readthedocs.io/en/stable/pipelines/>. [March 5, 2025].

19. **Bezgin G, Vakorin VA, van Opstal AJ, McIntosh AR, Bakker R**. Hundreds of brain maps in one atlas: registering coordinate-independent primate neuro-anatomical data to a standard brain. *Neuroimage* 62: 67-76, 2012. doi:10.1016/j.neuroimage.2012.04.013.

20. **Ramirez JSB**. Maternal nutrition and inflammation as a risk factor for future mental health disorders. Portland, OR: Oregon Health and Science University, 2019.

21. **Xu T, Nenning KH, Schwartz E, Hong SJ, Vogelstein JT, Goulas A, Fair DA, Schroeder CE, Margulies DS, Smallwood J, Milham MP, Langs G**. Cross-species functional alignment reveals evolutionary hierarchy within the connectome. *Neuroimage* 223: 117346, 2020. doi:10.1016/j.neuroimage.2020.117346.

22. **The MathWorks Inc.** MATLAB version: 9.12.0 (R2022a). Natick, Massachusetts, United States: The MathWorks Inc., 2022.

23. **Miranda-Dominguez O, Reiners P, Lee E, Carrasco CM, Conan G**. DCAN-Labs/biceps: BICEPS First release (beta) [Online]. Zenodo. doi.org/10.5281/zenodo.7697404. [March 3, 2023].

24. **Center for Developmental NeuroImaging (CDNI)**. DCAN-Labs/biceps: BICEPS First release [Online]. GitHub. <https://github.com/DCAN-Labs/biceps/releases/tag/beta>. [March 5, 2025].

25. **GUI_environments**. Introduction [Online]. Read the Docs. <https://gui-environments-documentation.readthedocs.io/en/latest/GUI_environments/>. [March 5, 2025].

[Page intentionally left blank]

# SUPPLEMENTAL TABLES S6 AND S7

**Table S6.** The 30 features with maximum variable importance values in the upper half of the observed range of values from the functional random forest (FRF) model of 4 month old macaque connectivity.

|  |  | ROI 1 | | | ROI 2 | | |
| --- | --- | --- | --- | --- | --- | --- | --- |
| Order of Importance | Maximum Variable Importance | Network | Hemi. | ROI Name | Network | Hemi. | ROI Name |
| 1 | 0.1925 | Somatomotor | Left | Primary Motor Cortex | Somatomotor | Left | Medial Premotor Cortex |
| 2 | 0.1588 | Somatomotor | Left | Secondary Somatosensory Cortex | Somatomotor | Right | Primary Motor Cortex |
| 3 | 0.1560 | Visual | Right | Visual Anterior Cortex, Dorsal Part | Somatomotor | Right | Medial Premotor Cortex |
| 4 | 0.1441 | Visual | Left | Visual Anterior Cortex, Ventral Part | Somatomotor | Right | Secondary Somatosensory Cortex |
| 5 | 0.1404 | Somatomotor | Left | Anterior Cingulate Gyrus | Somatomotor | Right | Primary Motor Cortex |
| 6 | 0.1360 | Auditory | Right | Secondary Auditory Cortex | Somatomotor | Right | Primary Somatosensory Cortex |
| 7 | 0.1359 | Visual | Left | Visual Area 2 | Visual | Right | Visual Area 1 |
| 8 | 0.1226 | Somatomotor | Left | Secondary Somatosensory Cortex | Somatomotor | Right | Medial Premotor Cortex |
| 9 | 0.1226 | Somatomotor | Left | Primary Somatosensory Cortex | Somatomotor | Right | Primary Motor Cortex |
| 10 | 0.1210 | Visual | Right | Visual Area 1 | Somatomotor | Left | Secondary Somatosensory Cortex |
| 11 | 0.1166 | Somatomotor | Left | Primary Somatosensory Cortex | Somatomotor | Left | Medial Premotor Cortex |
| 12 | 0.1134 | Visual | Right | Visual Anterior Cortex, Ventral Part | Somatomotor | Right | Primary Motor Cortex |
| 13 | 0.1124 | Somatomotor | Left | Primary Somatosensory Cortex | Somatomotor | Left | Primary Motor Cortex |
| 14 | 0.1106 | Visual | Right | Visual Anterior Cortex, Dorsal Part | Auditory | Right | Secondary Auditory Cortex |
| 15 | 0.1053 | Visual | Left | Visual Anterior Cortex, Ventral Part | Somatomotor | Left | Primary Somatosensory Cortex |
| 16 | 0.1042 | Visual | Right | Visual Area 1 | Somatomotor | Right | Posterior Insula |
| 17 | 0.1036 | Visual | Right | Visual Area 2 | Auditory | Left | Primary Auditory Cortex |
| 18 | 0.0994 | Visual | Right | Visual Area 2 | Somatomotor | Right | Posterior Insula |
| 19 | 0.0974 | Somatomotor | Right | Posterior Insula | Somatomotor | Right | Primary Somatosensory Cortex |
| 20 | 0.0957 | Visual | Left | Visual Anterior Cortex, Dorsal Part | Auditory | Left | Primary Auditory Cortex |
| 21 | 0.0942 | Visual | Right | Visual Anterior Cortex, Dorsal Part | Somatomotor | Right | Posterior Insula |
| 22 | 0.0926 | Visual | Left | Visual Area 1 | Somatomotor | Left | Medial Premotor Cortex |
| 23 | 0.0919 | Somatomotor | Left | Anterior Cingulate Gyrus | Somatomotor | Right | Medial Premotor Cortex |
| 24 | 0.0879 | Somatomotor | Left | Medial Premotor Cortex | Somatomotor | Right | Primary Motor Cortex |
| 25 | 0.0859 | Visual | Left | Visual Area 1 | Auditory | Left | Primary Auditory Cortex |
| 26 | 0.0841 | Somatomotor | Right | Primary Motor Cortex | Somatomotor | Right | Medial Premotor Cortex |
| 27 | 0.0823 | Somatomotor | Right | Secondary Somatosensory Cortex | Somatomotor | Right | Primary Motor Cortex |
| 28 | 0.0821 | Somatomotor | Left | Primary Somatosensory Cortex | Somatomotor | Right | Superior Parietal Cortex |
| 29 | 0.0811 | Visual | Left | Visual Area 2 | Auditory | Right | Primary Auditory Cortex |
| 30 | 0.0804 | Auditory | Left | Primary Auditory Cortex | Somatomotor | Left | Secondary Somatosensory Cortex |

Abbreviations: Hemi, brain hemisphere; ROI, region of interest.

**Table S7.** ROIs that were included in at least three of the 30 features with the greatest maximum variable importance from the functional random forest (FRF) model of 4 month old macaque connectivity.

| Network | Hemisphere | ROI Name | Number of Instances |
| --- | --- | --- | --- |
| Somatomotor | Right | Primary Motor Cortex | 7 |
| Somatomotor | Left | Primary Somatosensory Cortex | 5 |
| Auditory | Left | Primary Auditory Cortex | 4 |
| Somatomotor | Left | Medial Premotor Cortex | 4 |
| Somatomotor | Right | Medial Premotor Cortex | 4 |
| Somatomotor | Right | Posterior Insula | 4 |
| Somatomotor | Left | Secondary Somatosensory Cortex | 4 |
| Visual | Right | Visual Anterior Cortex, Dorsal Part | 3 |
| Visual | Right | Visual Area 1 | 3 |

The table is ordered first by the number of times an ROI appears in a feature, and then alphabetically by network and ROI name. Abbreviations: ROI, region of interest.

[Page intentionally left blank]

# SUPPLEMENTAL TABLE S8

**Table S8.** The 27 amygdala connections within the upper half of most important features from the functional random forest (FRF) model of 4 month old macaque connectivity.

|  |  | ROI 1 | | | ROI 2 | | |
| --- | --- | --- | --- | --- | --- | --- | --- |
| Order of Importance | Maximum Variable Importance | Network | Hemi. | ROI Name | Network | Hemi. | ROI Name |
| 44 | 0.0678 | Somatomotor | Left | Anterior Cingulate Gyrus | Limbic | Right | Amygdala |
| 47 | 0.0663 | Somatomotor | Right | Posterior Insula | Limbic | Left | Amygdala |
| 49 | 0.0653 | Somatomotor | Right | Medial Premotor Cortex | Limbic | Right | Amygdala |
| 50 | 0.0641 | Somatomotor | Left | Posterior Insula | Limbic | Right | Amygdala |
| 58 | 0.0592 | Somatomotor | Left | Primary Motor Cortex | Limbic | Left | Amygdala |
| 61 | 0.0583 | Somatomotor | Left | Posterior Insula | Limbic | Left | Amygdala |
| 62 | 0.0583 | Visual | Right | Visual Anterior Cortex, Ventral Part | Limbic | Right | Amygdala |
| 69 | 0.0561 | Visual | Right | Visual Anterior Cortex, Dorsal Part | Limbic | Right | Amygdala |
| 85 | 0.0519 | Visual | Right | Visual Area 1 | Limbic | Right | Amygdala |
| 86 | 0.0516 | Somatomotor | Right | Primary Somatosensory Cortex | Limbic | Left | Amygdala |
| 96 | 0.0491 | Visual | Right | Visual Area 2 | Limbic | Left | Amygdala |
| 109 | 0.0474 | Somatomotor | Left | Secondary Somatosensory Cortex | Limbic | Right | Amygdala |
| 119 | 0.0455 | Somatomotor | Right | Primary Motor Cortex | Limbic | Right | Amygdala |
| 121 | 0.0454 | Somatomotor | Right | Primary Motor Cortex | Limbic | Left | Amygdala |
| 124 | 0.0451 | Somatomotor | Right | Anterior Cingulate Gyrus | Limbic | Right | Amygdala |
| 138 | 0.0434 | Auditory | Left | Primary Auditory Cortex | Limbic | Right | Amygdala |
| 140 | 0.0430 | Somatomotor | Right | Anterior Cingulate Gyrus | Limbic | Left | Amygdala |
| 141 | 0.0430 | Somatomotor | Left | Primary Somatosensory Cortex | Limbic | Left | Amygdala |
| 146 | 0.0427 | Auditory | Right | Secondary Auditory Cortex | Limbic | Right | Amygdala |
| 147 | 0.0427 | Visual | Left | Visual Anterior Cortex, Dorsal Part | Limbic | Left | Amygdala |
| 148 | 0.0426 | Somatomotor | Right | Superior Parietal Cortex | Limbic | Right | Amygdala |
| 150 | 0.0424 | Auditory | Right | Primary Auditory Cortex | Limbic | Left | Amygdala |
| 152 | 0.0422 | Visual | Left | Visual Area 1 | Limbic | Right | Amygdala |
| 155 | 0.0418 | Somatomotor | Right | Superior Parietal Cortex | Limbic | Left | Amygdala |
| 157 | 0.0413 | Somatomotor | Left | Primary Motor Cortex | Limbic | Right | Amygdala |
| 160 | 0.0409 | Visual | Left | Visual Anterior Cortex, Ventral Part | Limbic | Left | Amygdala |
| 184 | 0.0369 | Somatomotor | Left | Anterior Cingulate Gyrus | Limbic | Left | Amygdala |

Abbreviations: Hemi, brain hemisphere; ROI, region of interest.
